# A comparative study of apoptosis, pyroptosis, necroptosis, and PANoptosis components in mouse and human cells

**DOI:** 10.1371/journal.pone.0299577

**Published:** 2024-02-27

**Authors:** Sk Mohiuddin Choudhury, Roman Sarkar, Rajendra Karki, Thirumala-Devi Kanneganti

**Affiliations:** Department of Immunology, St. Jude Children’s Research Hospital, Memphis, TN, United States of America; Toho University Graduate School of Medicine, JAPAN

## Abstract

Regulated cell death is a key component of the innate immune response, which provides the first line of defense against infection and homeostatic perturbations. However, cell death can also drive pathogenesis. The most well-defined cell death pathways can be categorized as nonlytic (apoptosis) and lytic (pyroptosis, necroptosis, and PANoptosis). While specific triggers are known to induce each of these cell death pathways, it is unclear whether all cell types express the cell death proteins required to activate these pathways. Here, we assessed the protein expression and compared the responses of immune and non-immune cells of human and mouse origin to canonical pyroptotic (LPS plus ATP), apoptotic (staurosporine), necroptotic (TNF-α plus z-VAD), and PANoptotic (influenza A virus infection) stimuli. When compared to fibroblasts, both mouse and human innate immune cells, macrophages, expressed higher levels of cell death proteins and activated cell death effectors more robustly, including caspase-1, gasdermins, caspase-8, and RIPKs, in response to specific stimuli. Our findings highlight the importance of considering the cell type when examining the mechanisms regulating inflammation and cell death. Improved understanding of the cell types that contain the machinery to execute different forms of cell death and their link to innate immune responses is critical to identify new strategies to target these pathways in specific cellular populations for the treatment of infectious diseases, inflammatory disorders, and cancer.

## Introduction

The innate immune system serves as the first line of defense against infections and sterile insults [1–3]. Membrane-bound and cytosolic pattern recognition receptors (PRRs) that sense pathogen-associated molecular patterns (PAMPs) and damage-associated molecular patterns (DAMPs) initiate the innate immune response and drive inflammatory signaling cascades that facilitate host defense [4–6]. In response to infections and stress, PRR sensing activates regulated cell death pathways, including non-lytic apoptosis and lytic pyroptosis, necroptosis, and PANoptosis. Apoptosis is defined as a non-lytic form of cell death activated by apoptotic caspases, caspases-8 and -9, and executed by executioner caspases-3 and -7. Among the lytic cell death pathways, pyroptosis is defined as a form of cell death that is activated by inflammatory caspases, including human and mouse caspase-1, human caspases-4 and -5, or mouse caspase-11, and executed by gasdermin D (GSDMD). Necroptosis is defined as a cell death pathway activated in the absence or upon inhibition of caspase-8 that acts via RIPK1/3 and is executed by MLKL. PANoptosis is defined as a unique innate immune inflammatory lytic cell death pathway driven by caspases and RIPKs that is regulated by PANoptosome complexes and executed by gasdermins, MLKL, and potentially other unknown proteins cleaved by caspases [7].

Molecularly, these cell death pathways are activated in response to distinct triggers and are executed by diverse protein complexes and executioners, making it critical to distinguish these pathways for therapeutic targeting. Each of these cell death pathways is controlled by multi-protein cell death complexes [7–12]. While inflammasome-dependent pyroptosis is characterized by the activation of inflammatory caspases and GSDMD for IL-1β and IL-18 maturation and cell lysis [8, 13–18], the intrinsic apoptosis pathway is mediated by intracellular stresses that lead to the formation of the apoptosome and activation of executioner caspases (caspases-3, -6, and -7) [9, 10, 19–21], extrinsic apoptosis involves the formation of complex II and the activation of executioner caspases [11, 22–24], and necroptosis is mediated by the necrosome through RIPK3 and the downstream effector MLKL in the absence or inhibition of caspase-8 [25–31]. In contrast to these classical cell death pathways, PANoptosis is characterized by activation of caspases and RIPKs and regulated by the multiprotein PANoptosome complexes [32–40]. PANoptosis plays an important role in innate immune responses and is associated with several infections, inflammatory diseases, and cancers [33, 34, 36–49]. The PANoptosis pathway shares many molecular components with the apoptosis, pyroptosis, and necroptosis pathways but has been consistently shown to be distinct [38–40]. To date, ZBP1, AIM2, RIPK1, and NLRP12 have been identified as essential sensors and regulators in the formation of different PANoptosome complexes [34, 43, 44, 49]. While various triggers can induce the formation of distinct cell death complexes (i.e., inflammasomes, apoptosomes, necrosomes, and PANoptosomes), it is unclear whether all cell types express the proteins required to execute each of these cell death mechanisms. To gain insight into the propensity of different cell types to trigger these unique cell death pathways, we examined the expression of various cell death markers in immune and non-immune cells from mice and humans. Our findings demonstrate that, when compared to non-immune cells, macrophages (both mouse and human) have greater basal expression, upregulation, or activation of essential cell death molecules such as caspase-1, gasdermins, caspase-8, and RIPKs, and they can respond to more diverse stimuli to trigger distinct cell death pathways. An in-depth understanding of the differences in cell death protein expression between cell types, as well as the roles for these cell type-specific differences in cell death for disease pathology, is critical to identifying therapeutic targets to modulate cell death pathways in specific cellular populations in infections, inflammation, and cancer.

## Materials and methods

### Generation of bone marrow-derived macrophages (BMDMs)

All work involving mice was reviewed and approved by the St. Jude IACUC Committee under protocol number 482. Primary bone marrow cells from wild-type (WT) mice were grown for 6 days in IMDM growth media containing IMDM (12440053, Thermo Fisher Scientific) supplemented with 10% heat-inactivated fetal bovine serum (HI-FBS; S1620, Biowest), 30% L929 conditioned media, 1% non-essential amino acids (11140–050, Thermo Fisher Scientific), and 1% penicillin and streptomycin (15070–063, Thermo Fisher Scientific). After 6 days, BMDMs were harvested, washed, and resuspended at a density of 1 × 10^6^ cells/mL in IMDM growth media. BMDMs were then seeded at a density of 1 × 10^6^ cells/well in 12-well plates or 5 × 10^5^ cells/well in 24-well plates in IMDM growth media overnight before use.

### Differentiation of human monocyte-derived macrophages (Hu-mΦ) and gene knock-down by siRNA transfection

Fresh human blood was collected from the apheresis rings of anonymous healthy blood donors from the blood bank of St. Jude Children’s Research Hospital (St. Jude), following a protocol reviewed and approved by the St. Jude IRB. Donors signed a standard written informed consent document that explains the apheresis process and important details prior to donating. Peripheral blood mononuclear cells (PBMCs) were isolated from the freshly collected blood using lymphoprep solution (07801/07811, Stemcell Technologies). Human monocytes were purified from PBMCs using a monocyte isolation kit (EasySep direct human monocyte isolation kit, 19669, Stemcell Technologies) strictly in accordance with the manufacturer’s protocol. The purified monocytes were further differentiated into monocyte-derived macrophages by culturing in RPMI growth media containing RPMI 1640 media (10-040-CV, Corning) supplemented with 10% HI-FBS, 1% penicillin and streptomycin, and 25 ng/mL human M-CSF (300–25, Peprotech) for 6 days in a CO_2_ incubator supplied with 5% CO_2_ in a humidified atmosphere. On days 2 and 4, an additional 8–10 mL of media containing 25 ng/mL human M-CSF was added to the cells. On day 6, all the loosely attached and suspension cells were harvested and washed three times with PBS. The cells were resuspended in RPMI growth media at a cell density of 1 × 10^6^ cells/mL. Hu-mΦ were then seeded at a density of 1 × 10^6^ cells/well in 12-well plates or 5 × 10^5^ cells/well in 24-well plates in RPMI growth media overnight before use.

For siRNA transfection, monocyte-derived macrophages were washed and resuspended at a cell density of 1 × 10^7^ cells/ml. A total of 5 pmoles of non-targeting control siRNA (D-001206-14-20, Horizon Discovery), human-specific *ZBP1* siRNA (M-014650-00-0005, Horizon Discovery), or human-specific *RIPK3* siRNA (M-003534-01-0005, Horizon Discovery) per million cells were used for transfection by electroporation (Neon Transfection System kit, MPK5000, Thermo Fisher Scientific).

### Cell lines

L929 cells were purchased from ATCC (CCL-1) and cultivated in DMEM (11995–065, Gibco) supplemented with 10% HI-FBS and 1% penicillin and streptomycin. Normal human lung fibroblasts (NHLF) were purchased from Lonza (CC-2512) and maintained in fibroblast growth medium (CC-3132, Lonza) containing 2% HI-FBS and 1% insulin, 1% human fibroblast growth factor (hFGF), and 1% gentamycin and amphotericin. L929 cells and NHLF cells were seeded at a density of 0.3 × 10^6^ cells/well in 12-well plates or 0.15 × 10^6^ cells/well in 24-well plates with respective growth media overnight before use for downstream experiments.

### Cell stimulation

BMDMs and L929 cells were stimulated in DMEM containing 10% HI-FBS and 1% penicillin and streptomycin. The human monocyte-derived macrophages and NHLF cells were stimulated in RPMI media supplemented with 10% HI-FBS and 1% penicillin and streptomycin, unless otherwise indicated. The following PAMPs, DAMPs, and inhibitors alone or in combinations were used as indicated: 100 ng/mL LPS (tlrl-3pelps, InvivoGen), 5 mM ATP (10127531001, Roche), 5 μM STS (S5921-1mg, Sigma-Aldrich), 2.5 μM Smac (S7009-LCL161, Selleckchem), 50 ng/mL TNF-α (315-01A, Peprotech), 25 μM Z-VAD(OMe)-FMK (z-VAD; 14463, Cayman Chemical), and 50 ng/mL IFN-γ (315–05, Peprotech (mouse) and 300–02, Peprotech (human)). A total of 500 μL or 250 μL of media with the indicated ligands was used for stimulation in 12- or 24-well plates, respectively. For LPS plus ATP stimulation, the mouse and human cells were preincubated with 100 ng/mL LPS for 4 h, followed by treatment with 5 mM ATP. The cell lysates were then collected 20 min post-ATP treatment from mouse cells and 1 h post-ATP treatment from human cells. For TNF-α plus z-VAD treatments, the mouse and human cells were preincubated with 25 μM z-VAD for 1 h, followed by stimulation with 50 ng/mL TNF-α. The cell lysates were then collected 6 h post–TNF-α addition from both mouse and human cells. For TSZ treatment, the human cells were preincubated with 25 μM z-VAD for 1 h, followed by stimulation with 50 ng/mL TNF-α plus 2.5 μM Smac. The cell lysates were then collected 6 h post–TNF-α and Smac addition. For STS treatment, both the mouse and human cell types were treated with 5 μM STS, and cell lysates were collected 2 h post-STS treatment. For IAV infection, we utilized a murine-adapted IAV strain (A/Puerto Rico/8/34 (H1N1) (PR8)) [50]. Both mouse and human cells were infected at an MOI of 20 in DMEM plain media (D6171, Sigma). After IAV infection for 1 h, cells were supplemented with 10% HI-FBS, and then cell lysates were collected 16 h post-IAV infection from both mouse and human cells.

### Real-time imaging for cell death

The kinetics of cell death were determined using the IncuCyte S3 (Sartorius) live-cell automated system. BMDMs (5 × 10^5^ cells/well) in 24-well tissue culture plates or (1 × 10^6^ cells/well) in 12-well tissue culture plates were treated with respective triggers and stained with propidium iodide (PI; P3566, Life Technologies) following the manufacturer’s protocol. The plate was scanned, and fluorescence and phase-contrast images (4 image fields per well) were acquired in real-time. PI-positive dead cells are marked with a red mask for visualization. The image analysis, masking, and quantification of dead cells were done using the software package supplied with the IncuCyte imager.

### Immunoblot analysis

For caspase blots, the cells were lysed along with the supernatant using 50 μL caspase lysis buffer [51] containing 5% NP-40 solution, 10 mM DTT, and 1× protease inhibitor solution. For signaling blots, supernatants were removed at the indicated timepoints, and cells were washed once with PBS, followed by cell lysis with RIPA buffer. SDS-PAGE electrophoresis was carried out to separate proteins on 8%−12% polyacrylamide gels. PVDF membranes were used to transfer the resolved proteins, and the blots were blocked with 5% skim milk for 1 h at room temperature. Blots were incubated with primary antibodies at 4°C overnight, followed by incubation with secondary HRP antibodies for 1 h at room temperature. The GE Amersham Imager 600 was used to image the immunoblots. In certain cases, the immunoblots were stripped using stripping buffer (Restore^TM^ Western Blot Stripping buffer, 21059, Thermo Fisher Scientific) and reprobed for another protein of interest.

Antibodies used were: anti-caspase-1 (AG-20B-0042, AdipoGen, 1:2000), human anti-caspase-1 (#3866, Cell Signaling Technologies [CST], 1:2000), human anti- cleaved caspase-1 (#4199, CST, 1:2000), anti-caspase-3 (#9662, CST, 1:1000), anti-cleaved caspase-3 (#9661, CST, 1:1000), anti-caspase-4 (#56056, SantaCruz, 1:1000), anti-caspase-7 (#9492, CST, 1:1000), anti-cleaved caspase-7 (#9491, CST, 1:1000), anti-caspase-8 (#4927, CST, 1:1000), anti-cleaved caspase-8 (#8592, CST, 1:1000), human anti-caspase-8 (ALX-804-242, Enzo Life Science, 1:1000), anti-caspase-11 (NB120-10454, Novus Biologicals, 1:1000), anti-caspase-6 (#9762, CST, 1:1000), anti-caspase-9 (#9504, CST, 1:1000), anti-AIM2 (ab119791, Abcam, 1:1000), anti-pMLKL (#37333, CST,1:1000), human anti-pMLKL (ab187091 (S-358), Abcam, 1:1000), anti-GSDMD (ab209845, Abcam, 1:1000), human anti-GSDMD (NBP2-33422, Novus Biologicals, 1:2000), anti-GSDME (ab215191, Abcam, 1:1000), anti-ASC (AG-25B-0006, AdipoGen, 1:2000), anti-IRF1 (#8478, CST, 1:1000), anti-MLKL (AP14272b, Abgent, 1:1000), human anti-MLKL (M6697, Sigma, 1:1000), anti-NLRP3 (AG-20B-0014, AdipoGen, 1:1000), anti-RIPK1 (#3493, CST, 1:1000), mouse anti-pRIPK3 (#91702S, CST, 1:1000), human anti-RIPK3 (#13526S, CST, 1:1000), human anti-pRIPK3 (ab209384, Abcam, 1:1000), anti-RIPK3 (2283, ProSci, 1:1000), anti-ZBP1 (AG-20B-0010, AdipoGen, 1:1000), human anti-ZBP1 (271483, SantaCruz, 1:1000), human anti-caspase-9 (#9502, CST, 1:1000), anti-GAPDH (#5174, CST, 1:5000), anti-β-actin (66009-1-IG, Proteintech, 1:5000), and HRP-conjugated secondary antibodies (anti-rabbit [111-035-047], 1:5000; anti-rat [112-095-003], 1:5000; and anti-mouse [315-035-047], 1:5000, Jackson ImmunoResearch Laboratories). Antibodies for pro- and cleaved forms of caspases were added together to the same blot to visualize both forms of the protein together, as reported previously [51].

## Results

### Innate immune cells are highly susceptible to multiple lytic cell death pathways compared with non-immune cells

**Fig 1:**
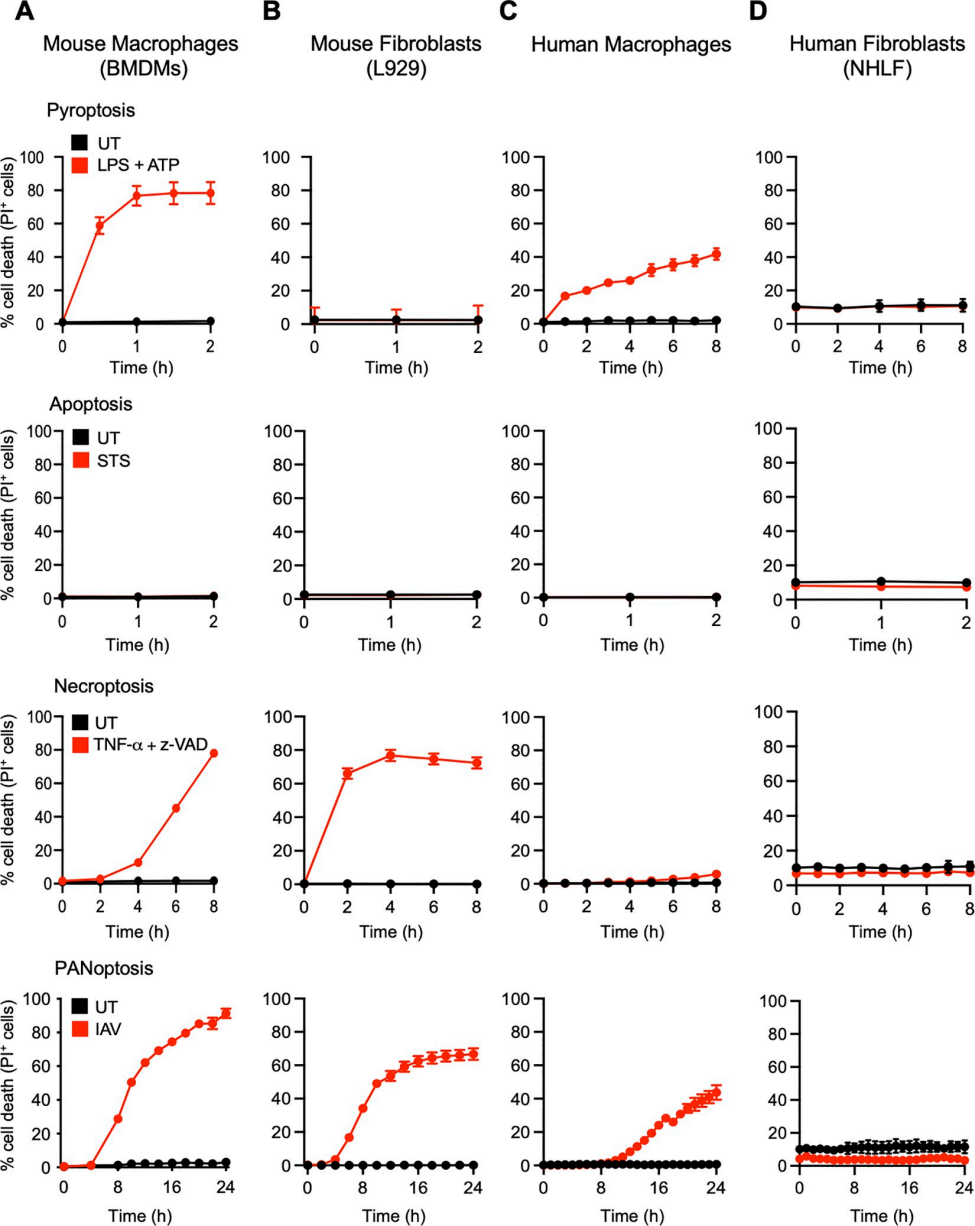
Differing susceptibility of immune and non-immune cells to diverse cell death triggers. (A-D) Real-time cell death analysis in untreated cells (UT; black curves) vs cells treated with cell death triggers (red curves), including lipopolysaccharide (LPS) plus ATP, staurosporine (STS), TNF-α plus z-VAD, or influenza A virus (IAV), in (A) mouse bone marrow-derived macrophages (BMDMs), (B) mouse fibroblasts (L929 cells), (C) human macrophages, and (D) normal human lung fibroblasts (NHLF cells). The data are shown as mean ± SEM and are representative of at least three independent biological replicates.

Regulated cell death plays a central role throughout the body in homeostasis, host defense, inflammation, and disease pathology [4–7]. Depending on the molecular machinery involved, cell death can occur in trigger- and time-specific manners. However, the ability of distinct cell types to undergo different forms of cell death is not well understood. To determine whether specific triggers can activate apoptotic, pyroptotic, necroptotic, or PANoptotic cell death in human and mouse innate immune cells (macrophages) and non-immune cells (fibroblasts), we treated murine bone marrow-derived macrophages (BMDMs) and L929 cells (murine fibroblasts), as well as human macrophages and human lung fibroblasts (NHLF cells), with LPS plus ATP (pyroptotic trigger), staurosporine (STS; apoptotic trigger), TNF-α plus z-VAD (necroptotic trigger), or influenza A virus (IAV; PANoptotic trigger), and assessed lytic cell death **(Fig 1A–1D)**. In response to the canonical pyroptotic inflammasome trigger LPS priming and ATP stimulation, BMDMs demonstrated significant, rapid cell death, but L929 cells were resistant to lytic cell death even at 2 h post-treatment **(Fig 1A and 1B)**. Furthermore, no substantial lytic cell death was observed in BMDMs or L929 cells within 2 h of STS treatment **(Fig 1A and 1B)**. STS is known to elicit rapid, non-lytic, apoptotic cell death [52]. Therefore, the 2 h timepoint was selected in our study to observe the canonical activation of apoptosis. As apoptosis is a non-lytic cell death pathway in which the cell membrane remains intact, we did not observe any PI uptake at the 2 h timepoint post-STS treatment **(Fig 1A and 1B)**, while non-lytic cell death is expected to be occurring at this timepoint. In contrast, both BMDMs and L929 cells underwent lytic cell death in response to TNF-α plus z-VAD treatment and IAV infection, with BMDMs demonstrating slightly higher levels of cell death compared to the fibroblasts at late timepoints **(Fig 1A and 1B)**. Similarly, we observed that human macrophages were more susceptible to cell death in response to LPS plus ATP treatment compared to NHLF cells, with macrophages beginning to undergo cell death within 1 h but NHLF cells remaining resistant to cell death through 8 h of treatment. However, prolonged exposure to LPS plus ATP treatment in NHLF cells induced lytic cell death after 24 h, which plateaued after 30 h of treatment **(S1 Fig)**. Furthermore, no significant lytic cell death was observed in either of these human cell types within 2 h of STS treatment **(Fig 1C and 1D)**. The necroptotic trigger TNF-α plus z-VAD did not induce cell death in both human macrophages and fibroblasts. Therefore, we added Smac (second mitochondrial activator of caspases) to the necroptotic trigger, as this is known to induce necroptosis in human cells [53]. The combination of TNF-α, z-VAD, and Smac (TSZ) induced cell death in human macrophages but not in NHLF fibroblasts **(S2 Fig)**. IAV treatment induced significant cell death in human macrophages, whereas no cell death was observed in NHLF cells within 24 h following treatment **(Fig 1C and 1D)**. These findings suggest that immune and non-immune cells respond differently to diverse stimuli, and that immune cells are likely to be more susceptible to innate immune-mediated cell death pathways.

### Innate immune cells express an array of cell death proteins, whereas non-immune cells have a relatively limited repertoire

**Fig 2:**
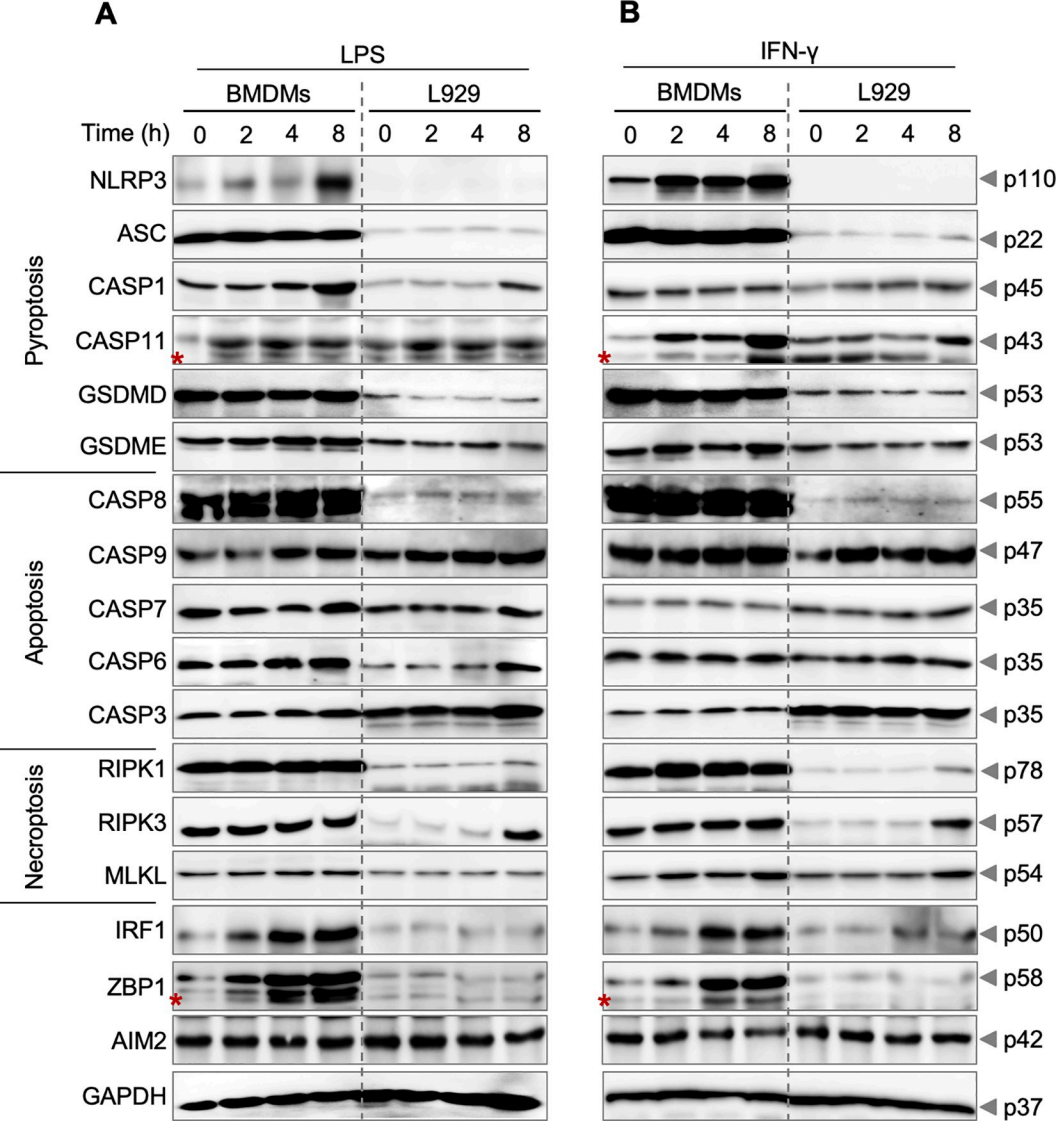
Cell death proteins are differentially expressed in mouse cell types. (A, B) Immunoblot analysis of different cell death proteins in mouse bone marrow-derived macrophages (BMDMs) and L929 cells at different time points (0, 2, 4, and 8 h) following (A) lipopolysaccharide (LPS) or (B) IFN-γ treatment. Different cell death proteins were assessed by immunoblotting for pyroptosis indicators: NLRP3 (p110), ASC (p22), caspase-1 (CASP1; p45), caspase-11 (CASP11; p43), gasdermin D (GSDMD; p53), and gasdermin E (GSDME; p53); apoptosis indicators: caspase-8 (CASP8; p55), caspase-9 (CASP9; p47), caspase-7 (CASP7; p35), caspase-6 (CASP6; p35), and caspase-3 (CASP3; p35); necroptosis indicators: receptor-interacting protein kinase 1 (RIPK1; p78), receptor-interacting protein kinase 3 (RIPK3; p57), and mixed lineage kinase domain-like pseudokinase (MLKL; p54); and PANoptosis regulators: interferon regulatory factor 1 (IRF1; p50), Z-DNA binding protein 1 (ZBP1; p58), and absent in melanoma 2 (AIM2; p42). GAPDH was used as a loading control. The data are representative of at least three independent biological replicates, with the most representative blot for each protein being selected from across the experiments. We verified comparable protein loading between experiments by comparing GAPDH blots. The asterisks indicate non-specific bands.

We next examined the expression levels of various cell death proteins associated with pyroptosis, apoptosis, necroptosis, and PANoptosis cell death pathways in mouse and human immune and non-immune cells. We focused on these molecules because they are critical regulators and effectors of specific cell death pathways, and disruptions in their expression and functions can lead to the development of disease [54–56]. To obtain insight into the expression of key components of cell death in murine cell types, BMDMs and L929 cells were treated with LPS **(Fig 2A and S1 Appendix)** or IFN-γ **(Fig 2B and S1 Appendix)**, which are well-known inducers of inflammation and cell death. Overall, BMDMs had greater baseline expression of multiple cell death markers implicated in the activation of several cell death pathways (including PANoptosis, pyroptosis, apoptosis, and necroptosis) than L929 cells **(Fig 2A, 2B and S1 Appendix)**. In response to LPS stimulation, there was more induction of NLRP3, caspases-1 and -6, IRF1, and ZBP1 expression in BMDMs compared to L929 fibroblast. While the expression of ASC, GSDMD, GSDME, caspase-8, MLKL, and RIPK1 was higher in BMDMs compared to L929 cells, their expression remained only marginally induced or unchanged following LPS stimulation **(Fig 2A and S1 Appendix)**. Similarly, upon IFN-γ stimulation, BMDMs displayed a higher expression of NLRP3, caspase-11, RIPK1, IRF1, and ZBP1 compared to L929 fibroblasts. In contrast, the expression of ASC, GSDMD, GSDME, caspase-8, and RIPK3 was generally higher in BMDMs compared to L929 cells, and it remained unchanged following IFN-γ stimulation **(Fig 2B and S1 Appendix)**. In addition, following IFN-γ stimulation, both BMDMs and L929 fibroblasts exhibited a modest time-dependent increase in expression of caspase-9 and MLKL **(Fig 2B and S1 Appendix)**. The mouse fibroblasts had upregulated expression of caspase-1, IRF1, RIPK1, and RIPK3 at later time points (8 h) following treatment with IFN-γ **(Fig 2B and S1 Appendix)**. Taken together, these findings suggest that immune cells, in comparison to fibroblasts, are better equipped with key molecules that can contribute to the assembly of cell death complexes and the execution of diverse cell death pathways to respond to different stimuli.

**Fig 3:**
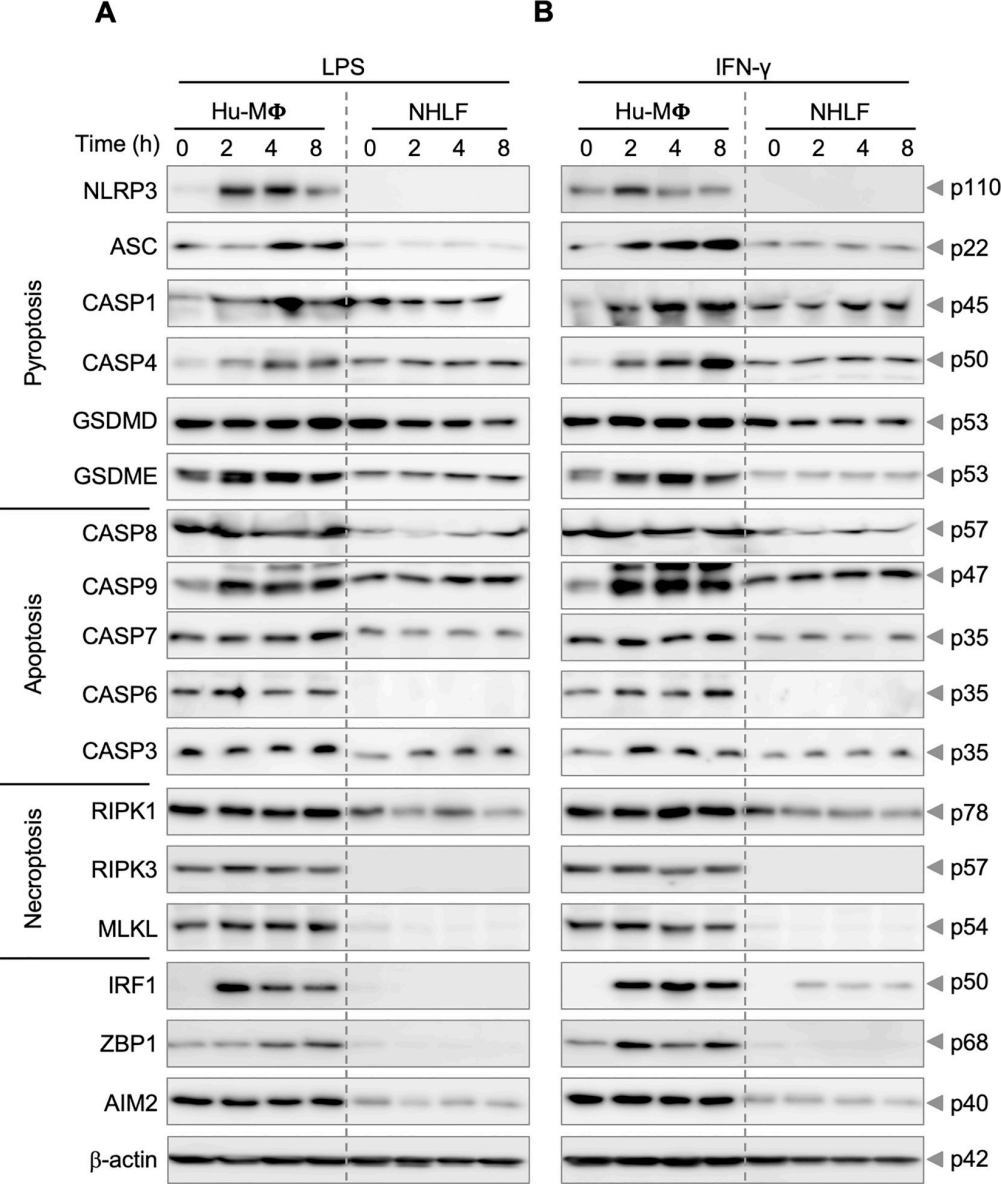
Cell death proteins are differentially expressed in human cell types. (A, B) Immunoblot analysis of different cell death proteins in human macrophages (Hu-Mϕ) and normal human lung fibroblasts (NHLF cells) at different time points (0, 2, 4, and 8 h) following (A) lipopolysaccharide (LPS) or (B) IFN-γ treatment. Different cell death proteins were assessed by immunoblotting for pyroptosis indicators: NLRP3 (p110), ASC (p22), caspase-1 (CASP1; p45), caspase-4 (CASP4; p50), gasdermin D (GSDMD; p53), and gasdermin E (GSDME; p53); apoptosis indicators: caspase-8 (CASP8; p57), caspase-9 (CASP9; p47), caspase-7 (CASP7; p35), caspase-6 (CASP6; p35), and caspase-3 (CASP3; p35); necroptosis indicators: receptor-interacting protein kinase 1 (RIPK1; p78), receptor-interacting protein kinase 3 (RIPK3; p57), and mixed lineage kinase domain-like pseudokinase (MLKL; p54); and PANoptosis regulators: interferon regulatory factor 1 (IRF1; p50), Z-DNA binding protein 1 (ZBP1; p68), and absent in melanoma 2 (AIM2; p40). β-actin was used as a loading control. The data are representative of at least three independent biological replicates, with the most representative blot for each protein being selected from across the experiments. We verified comparable protein loading between experiments by comparing β-actin blots.

Similarly, we compared the expression of various molecular components involved in different cell death pathways in primary human macrophages and NHLF cells following treatment with LPS **(Fig 3A and S1 Appendix)** or IFN-γ **(Fig 3B and S1 Appendix)**. Human macrophages expressed all the key cell death molecules assessed and showed upregulation of many of the molecules, such as caspases-1, -4, -9, and IRF1 in response to LPS or IFN-γ; in contrast, NHLF cells did not express many of the cell death molecules at detectable levels at baseline or in response to stimulation (e.g., NLRP3, caspase-6, RIPK3, MLKL, and ZBP1) **(Fig 3 and S1 Appendix)**. We noted that ZBP1 in human macrophages was identified as a higher molecular weight band than that observed in murine cells (**Figs 2, 3 and S1 Appendix**). To further validate this observation, we confirmed the specificity of the ZBP1 antibody using siRNA-mediated knockdown of *ZBP1* in human macrophages from two donors **(S3 Fig)**.

In contrast to the differences observed between human macrophages and NHLF cells, GSDMD expression levels were comparable between the two cell types following treatment with either LPS or IFN-γ. These findings suggest that human macrophages exhibit higher baseline expression of numerous components implicated in cell death pathways, indicating that they may be more likely to undergo innate immune cell death.

### Immune and non-immune cells undergo differential activation of cell death pathways in response to specific triggers

**Fig 4:**
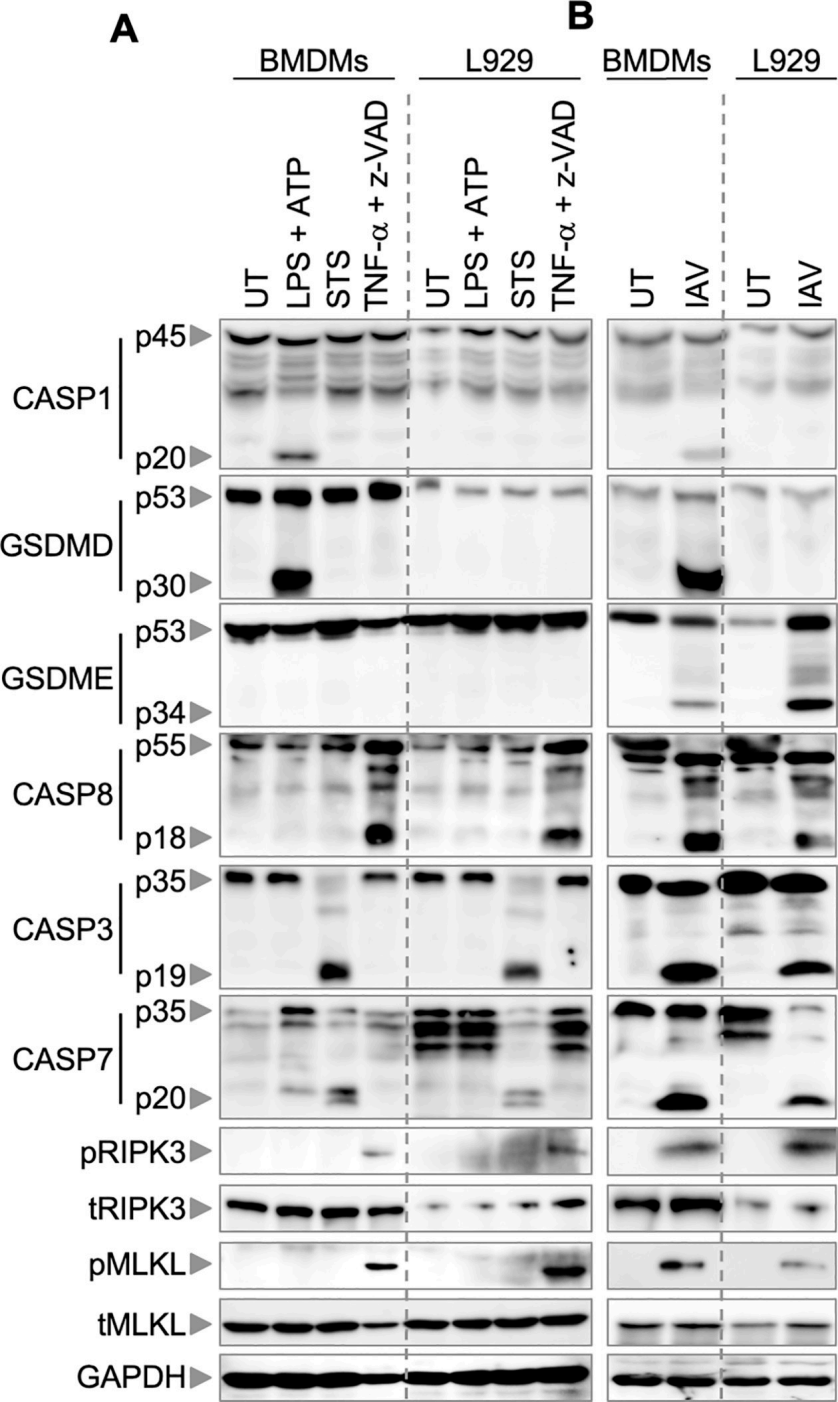
Trigger-specific activation of cell death components in mouse immune and non-immune cells. (A, B) Immunoblot analysis of cell death molecules in mouse bone marrow-derived macrophages (BMDMs) and L929 cells in untreated (UT) cells and following treatment with (A) lipopolysaccharide (LPS) plus ATP, staurosporine (STS), TNF-α plus z-VAD, or (B) influenza A virus (IAV). Activation of cell death molecules was assessed by monitoring cleavage of caspase-1 (CASP1; p20), gasdermin D (GSDMD; p30), gasdermin E (GSDME; p34), caspase-8 (CASP8; p18), caspase-3 (CASP3; p19), and caspase-7 (CASP7; p20), as well as phosphorylation of receptor-interacting protein kinase 3 (pRIPK3) and phosphorylation of mixed lineage kinase domain-like pseudokinase (pMLKL). Total RIPK3 (tRIPK3), total MLKL (tMLKL), and GAPDH were used as loading controls. The data are representative of at least three independent biological replicates, with the most representative blot for each protein being selected from across the experiments. We verified comparable protein loading between experiments by comparing GAPDH blots.

**Fig 5:**
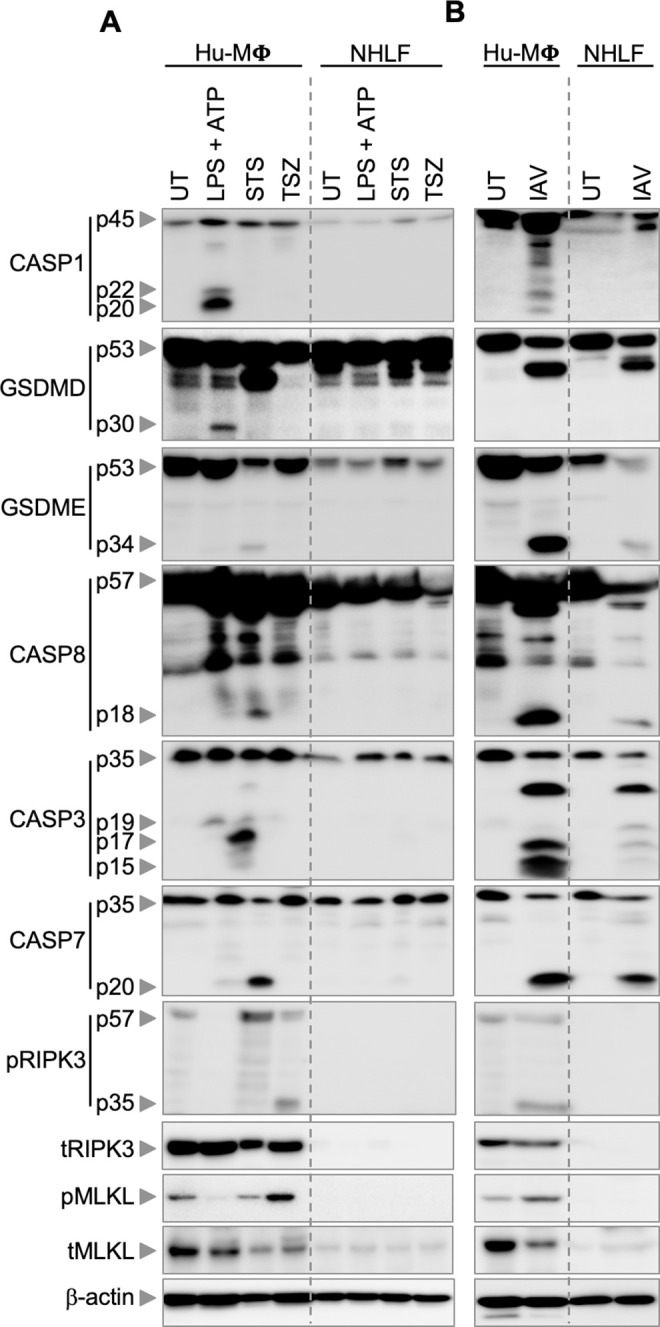
Trigger-specific activation of cell death components in human immune and non-immune cells. (A, B) Immunoblot analysis of cell death molecules in human macrophages (Hu-Mϕ) and normal human lung fibroblasts (NHLF cells) in untreated (UT) cells and following treatment with (A) lipopolysaccharide (LPS) plus ATP, staurosporine (STS), TNF-α plus z-VAD plus Smac (TSZ), or (B) influenza A virus (IAV). Activation of cell death molecules was assessed by monitoring cleavage of caspase-1 (CASP1; p22/20), gasdermin D (GSDMD; p30), gasdermin E (GSDME; p34), caspase-8 (CASP8; p18), caspase-3 (CASP3; p19/17/15), and caspase-7 (CASP7; p20), as well as phosphorylation of receptor-interacting protein kinase 3 (pRIPK3 (p57/35) and phosphorylation of mixed lineage kinase domain-like pseudokinase (pMLKL). Total MLKL (tMLKL), total RIPK3 (tRIPK3), and β-actin were used as loading controls. The data are representative of at least three independent biological replicates, with the most representative blot for each protein being selected from across the experiments. We verified comparable protein loading between experiments by comparing β-actin blots.

Given the contrasting cell death outcomes we observed in immune and non-immune cells when treated with specific pyroptotic, apoptotic, necroptotic, and PANoptotic triggers **(Fig 1)**, we next examined the expression and activation of different cell death molecules in murine and human immune and non-immune cells in response to these triggers **(Figs 4, 5 and S1 Appendix)**. Upon treatment with the canonical pyroptotic trigger, LPS plus ATP, we observed activation of caspase-1 and GSDMD in BMDMs but not in L929 cells **(Fig 4A and S1 Appendix)**. In response to the apoptotic trigger STS, caspase-3 and caspase-7 were activated in both BMDMs and L929 cells at 2 h post-treatment **(Fig 4A and S1 Appendix)**. Similarly, the necroptotic trigger TNF-α plus z-VAD resulted in caspase-8 cleavage and phosphorylation of RIPK3 (pRIPK3) and MLKL (pMLKL) in both BMDMs and L929 cells **(Fig 4A and S1 Appendix)**. Prior studies suggest that caspase-8 can be cleaved in response to the necroptotic trigger z-VAD [57], which is consistent with our observations. While we did not observe the activation of downstream apoptotic caspases such as caspases-3 and -7 in response to TNF-α plus z-VAD, we did observe the activation of necroptotic markers such as RIPK3 and MLKL **(Fig 4A and S1 Appendix)**, indicating that the observed caspase-8 cleavage may regulate necroptosis but is nonfunctional in inducing apoptosis. Furthermore, when treated with the PANoptosis trigger IAV, caspase-1 and GSDMD were activated in BMDMs but not in L929 cells **(Fig 4B and S1 Appendix)**, while GSDME, caspases-8, -3, and -7, pRIPK3, and pMLKL were activated in both immune and non-immune cells **(Fig 4B and S1 Appendix)**.

We next investigated how human macrophages and human lung fibroblasts responded to these specific cell death triggers **(Fig 5 and S1 Appendix)**. Human macrophages exhibited the activation of caspase-1 and GSDMD, along with low-level activation of caspase-3 (p19 only), caspase-7, and caspase-8 in response to the pyroptotic trigger LPS plus ATP, while NHLF cells did not **(Fig 5A and S1 Appendix)**. In contrast, neither cell type showed activation of pRIPK3 or pMLKL in response to LPS plus ATP treatment **(Fig 5A and S1 Appendix)**. The apoptotic trigger STS activated caspases-3, -7, and -8, and elevated levels of pRIPK3 in human macrophages, while only a low level of activation of caspase-7 was observed in NHLF cells **(Fig 5A and S1 Appendix)**. Phosphorylation of RIPK3 in response to STS in human macrophages was unexpected, and the mechanism remains to be established. We also observed a low level of GSDME cleavage in human macrophages in response to STS, which is known to be a result of caspase-3 activation in context-dependent manners [58, 59], but this level of cleavage appears insufficient to induce lysis, as we did not observe lytic cell death in response to STS at this timepoint (**Fig 1C**).

We observed that the necroptotic trigger TSZ induced cleavage of caspase-8 and activation of pMLKL in human macrophages but not in NHLF cells **(Fig 5A and S1 Appendix)**. We also observed a cleaved band for pRIPK3 (p35) in response to TSZ. We further confirmed that this p35 band was indeed RIPK3 by using siRNA-mediated knockdown of *RIPK3* in human macrophages **(S4 Fig)**. However, the mechanism that phosphorylates RIPK3 in response to TSZ is still unknown and requires further investigation. We also observed basal expression of pRIPK3 and pMLKL in untreated human macrophages.

In response to IAV, human macrophages showed activation of caspase-1, whereas GSDME and caspases-3, -8, and -7 were cleaved in both cell types **(Fig 5B and S1 Appendix)**. Moreover, the IAV-induced phosphorylation of RIPK3 (p35) and MLKL was only observed in human macrophages and not in NHLF fibroblasts **(Fig 5B and S1 Appendix)**. These findings highlight that immune and non-immune cells respond differently to diverse cell death triggers, which contributes to our understanding of how different cell types activate distinct protein complexes driving cell death.

## Discussion

Recent breakthroughs in the study of innate immune cell death pathways, including the discovery of PANoptosis and the characterization of central cell death regulators, have opened new avenues for developing innovative treatment strategies against a range of diseases. Several bacterial and viral infections, as well as sterile stimuli, can activate cell death pathways, including pyroptosis, apoptosis, necroptosis, and PANoptosis [6, 7, 19, 22, 25, 29, 33, 34, 36–49, 60]. Innate immune cells, such as macrophages, serve as the first responders to these stimuli, orchestrating finely tuned immune responses [2, 3, 6, 61, 62]. In the current study, we investigated how macrophages, as a model for immune cells, and fibroblasts, as a model for non-immune cells, activate cell death proteins to respond to diverse stimuli, thereby initiating and executing distinct cell death pathways. Our findings shed light on the fact that immune and non-immune cells exhibited differential cell death responses following exposure to various stimuli. We specifically observed that both human and murine macrophages were more susceptible to LPS plus ATP- and IAV-induced cell death when compared to non-immune cells. Moreover, human macrophages exhibited a high susceptibility to TSZ-induced cell death, whereas human fibroblasts were resistant. Further examination showed that the immune cells compared to non-immune cells demonstrated higher expression levels of key components of cell death complexes, such as NLRP3, ZBP1, ASC, caspase-1, caspase-8, and RIPK3, in response to LPS and IFN-γ. These findings suggest that immune cells, such as macrophages, possess the capability to activate cell death pathways that fibroblasts do not. Furthermore, in addition to the cell death complex molecules and regulators we assessed here, the expression of upstream receptors, including the cell surface TLRs, may also play a role in driving differential responses. For example, TLR4 is the main cell surface receptor responsible for sensing and responding to LPS [63], and its expression is often essential for initiating cell death downstream of LPS exposure. TLR4 is known to be expressed on immune cells, but its expression on non-immune cells is not ubiquitous. While TLR4 is expressed by human lung fibroblasts [64], there is no evidence that it is expressed by L929 cells. As a result, while NHLF fibroblasts might have the ability to undergo pyroptosis at late timepoints, it is unlikely that L929 cells will. These findings denote differences between cell types even within the fibroblast classification, highlighting the need to consider the specifics of cell types when characterizing cellular populations.

Moreover, the ability of macrophages to respond to infections and sterile triggers by undergoing lytic cell death and releasing pro-inflammatory cytokines and DAMPs is critical to their ability to alert the surrounding cells of the threat and initiate inflammatory and immune responses for host defense against infections and diseases. This may explain why macrophages are better equipped with cell death proteins and respond more precisely to specific triggers. This distinction may contribute to the increased immunological responsiveness and engagement of immune cells in different cell death pathways.

Overall, our study underscores the critical role played by innate immune cells in the regulation of innate immune responses and disease pathogenesis. The innate immune cells have crucial molecular components required for the formation of cell death complexes, enabling them to efficiently execute cell death in response to a variety of stimuli.

## Supporting information

S1 Fig Prolonged exposure to LPS plus ATP induces lytic cell death in NHLF cells. Real-time cell death analysis of untreated (black curve) and lipopolysaccharide (LPS) plus ATP-treated (red curve) normal human lung fibroblasts (NHLF cells) seeded in a 24-well plate at 0.15 x 10^6^ cells per well. After overnight culture, these cells were primed with LPS for 4 h, then treated with 5 mM ATP. The data are shown as mean ± SEM and are representative of at least three independent biological replicates.
(TIF)

S2 Fig Canonical necroptosis trigger induces lytic cell death in human macrophages. Real-time cell death analysis of untreated (black) and TNF-α plus z-VAD plus Smac (TSZ)-treated (red) human macrophages and normal human lung fibroblasts (NHLF cells). The data are shown as mean ± SEM and are representative of at least three independent biological replicates.
(TIF)

S3 Fig siRNA-mediated knockdown of *ZBP1* reduces expression in human macrophages. Immunoblot analysis of Z-DNA binding protein 1 (ZBP1; p68) expression in human macrophages treated with non-targeting siRNA (siControl) or human-specific *ZBP1* siRNA (si*ZBP1*). β-actin was used as a loading control. The data are representative of at least two independent experiments.
(TIF)

S4 Fig siRNA-mediated knockdown of *RIPK3* reduces expression in human macrophages. Immunoblot analysis of phosphorylated receptor-interacting protein kinase 3 (pRIPK3) and total RIPK3 (tRIPK3) expression in human macrophages treated with non-targeting siRNA (siControl) or human-specific *RIPK3* siRNA (si*RIPK3*), followed by treatment with TNF-α plus z-VAD plus Smac (TSZ). β-actin was used as a loading control. The data are representative of at least two independent biological replicates.
(TIF)

S1 Appendix Uncropped blots for immunoblot analyses. (PDF)

S1 Data (XLSX)

S2 Data (XLSX)

S3 Data (XLSX)
