# Supplementary figures and images for: A comparative study of apoptosis, pyroptosis, necroptosis, and PANoptosis components in mouse and human cells
Source: PLoS One. 2024 Feb 27;19(2):e0299577. doi: 10.1371/journal.pone.0299577 (PMC10898734; doi:10.1371/journal.pone.0299577)

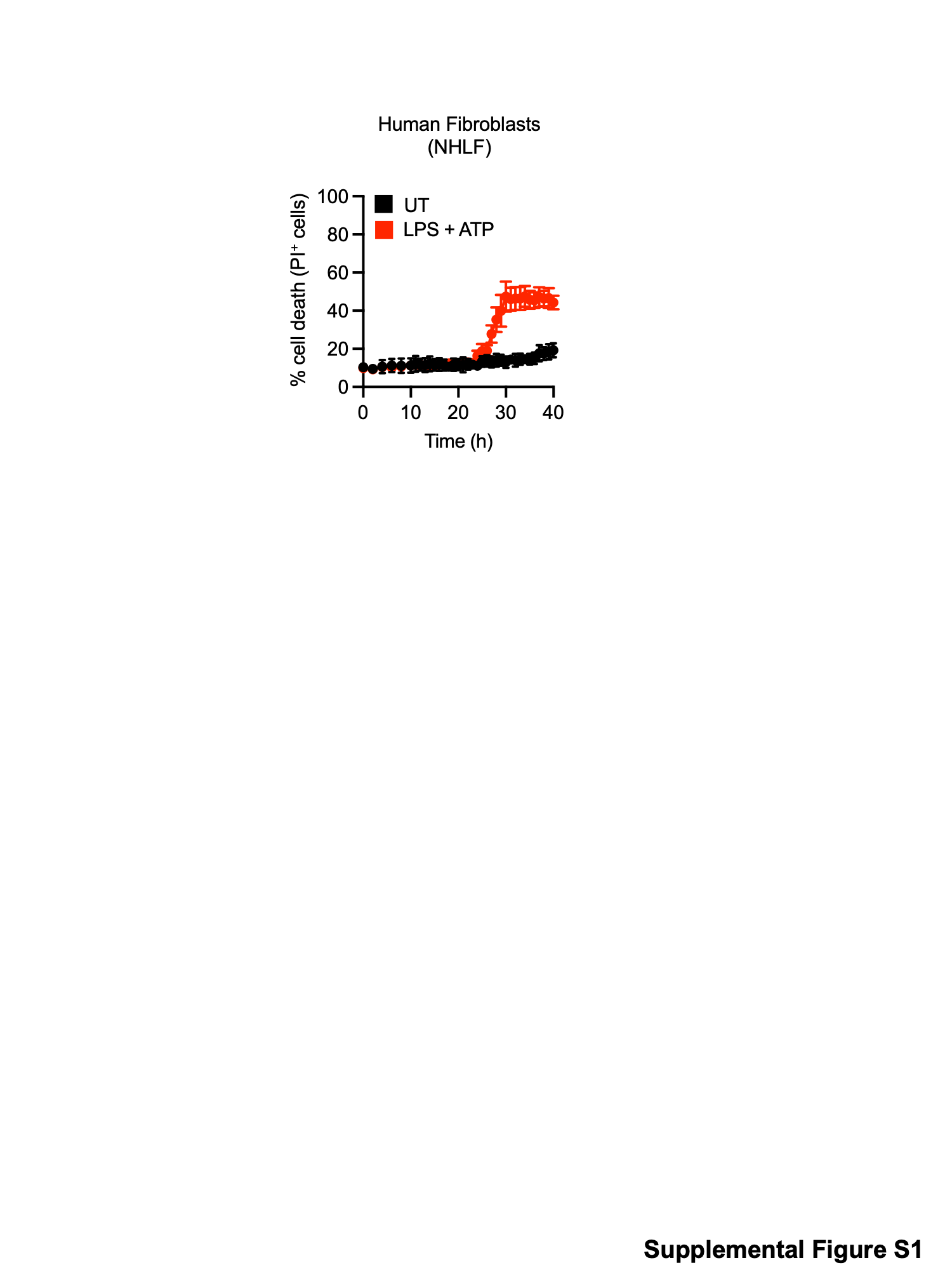

Supplement: S1 Fig — Real-time cell death analysis of untreated (black curve) and lipopolysaccharide (LPS) plus ATP-treated (red curve) normal human lung fibroblasts (NHLF cells) seeded in a 24-well plate at 0.15 x 10^6^ cells per well. After overnight culture, these cells were primed with LPS for 4 h, then treated with 5 mM ATP. The data are shown as mean ± SEM and are representative of at least three independent biological replicates. (TIF) [file pone.0299577.s001.tif]

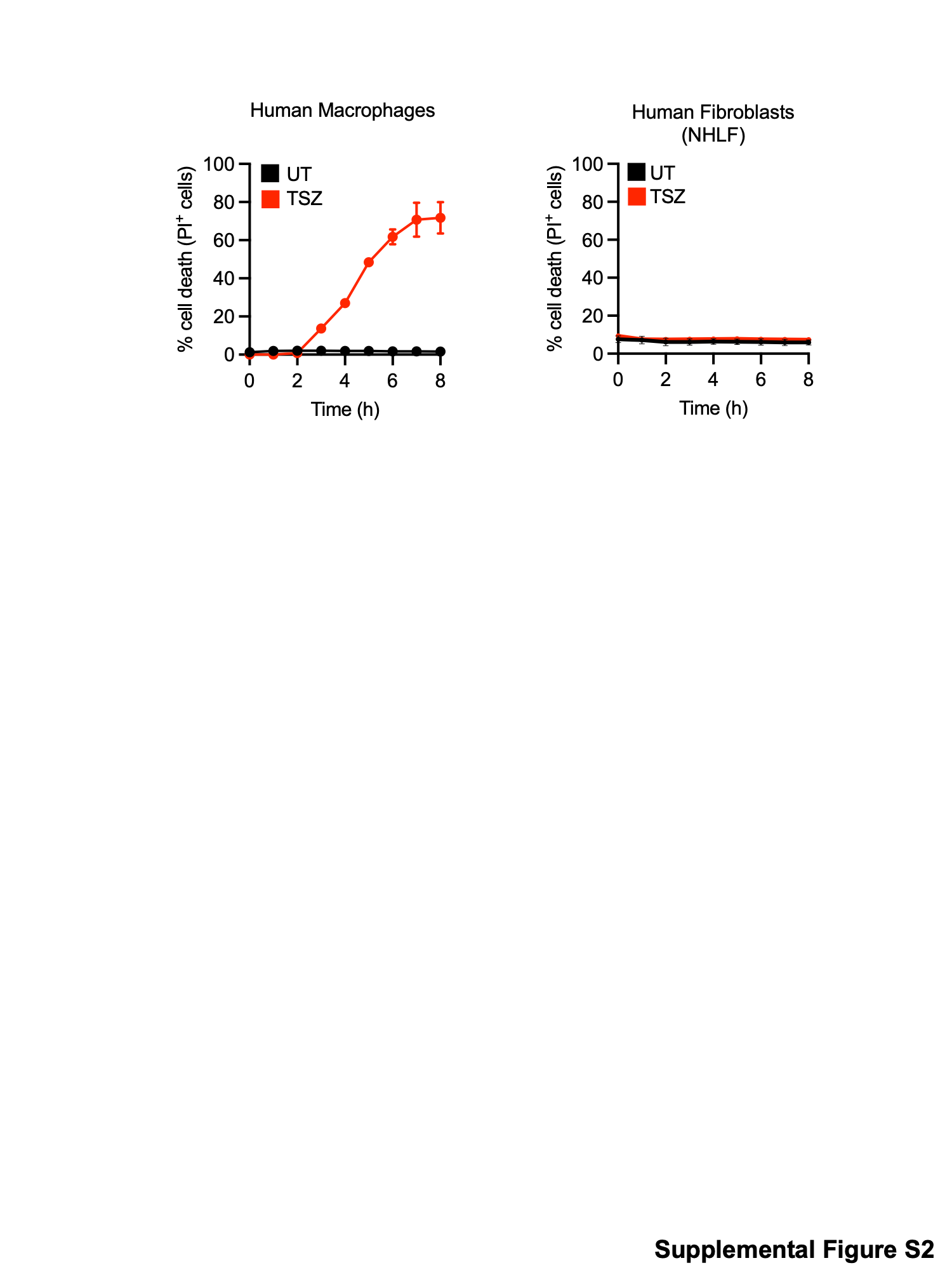

Supplement: S2 Fig — Real-time cell death analysis of untreated (black) and TNF-α plus z-VAD plus Smac (TSZ)-treated (red) human macrophages and normal human lung fibroblasts (NHLF cells). The data are shown as mean ± SEM and are representative of at least three independent biological replicates. (TIF) [file pone.0299577.s002.tif]

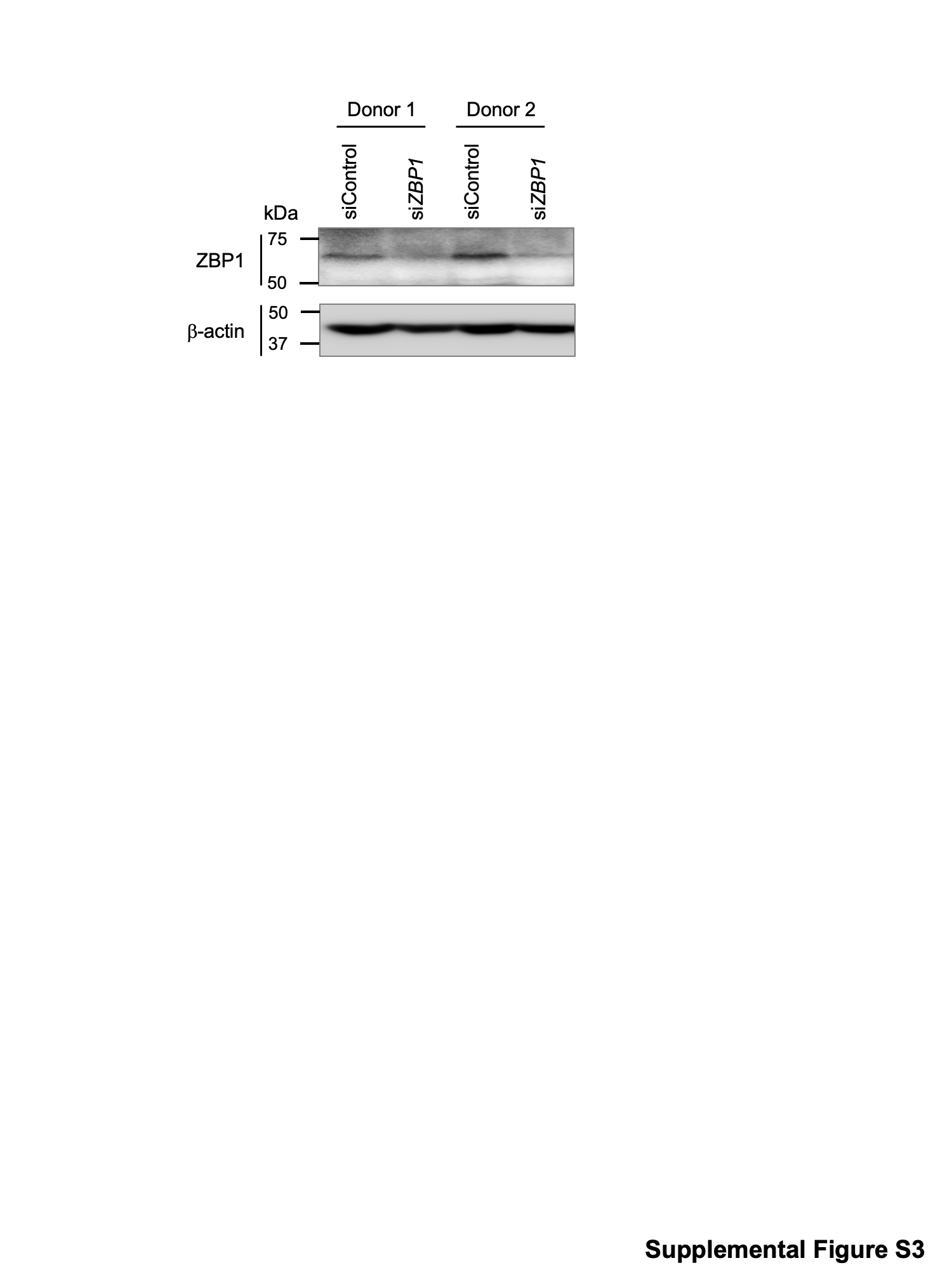

Supplement: S3 Fig — Immunoblot analysis of Z-DNA binding protein 1 (ZBP1; p68) expression in human macrophages treated with non-targeting siRNA (siControl) or human-specific ZBP1 siRNA (siZBP1). β-actin was used as a loading control. The data are representative of at least two independent experiments. (TIF) [file pone.0299577.s003.tif]

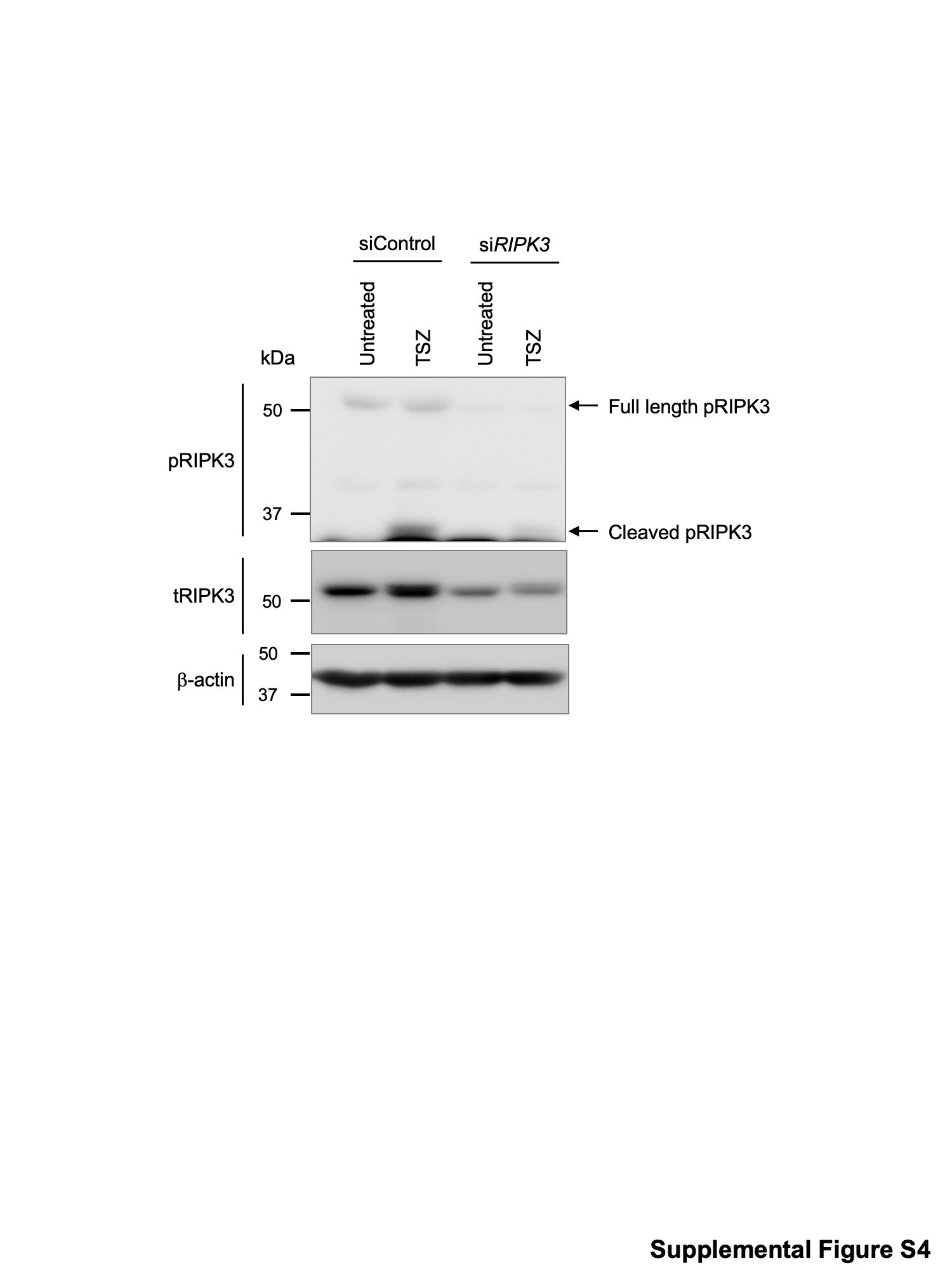

Supplement: S4 Fig — Immunoblot analysis of phosphorylated receptor-interacting protein kinase 3 (pRIPK3) and total RIPK3 (tRIPK3) expression in human macrophages treated with non-targeting siRNA (siControl) or human-specific RIPK3 siRNA (siRIPK3), followed by treatment with TNF-α plus z-VAD plus Smac (TSZ). β-actin was used as a loading control. The data are representative of at least two independent biological replicates. (TIF) [file pone.0299577.s004.tif]
