# Supplementary material for: A comparative study of apoptosis, pyroptosis, necroptosis, and PANoptosis components in mouse and human cells
Source: PLoS One. 2024 Feb 27;19(2):e0299577. doi: 10.1371/journal.pone.0299577 (PMC10898734; doi:10.1371/journal.pone.0299577)

Appendix 1. Uncropped blots for immunoblot analyses.

Figure 2 uncropped blots (pages 1-6):

NLRP3

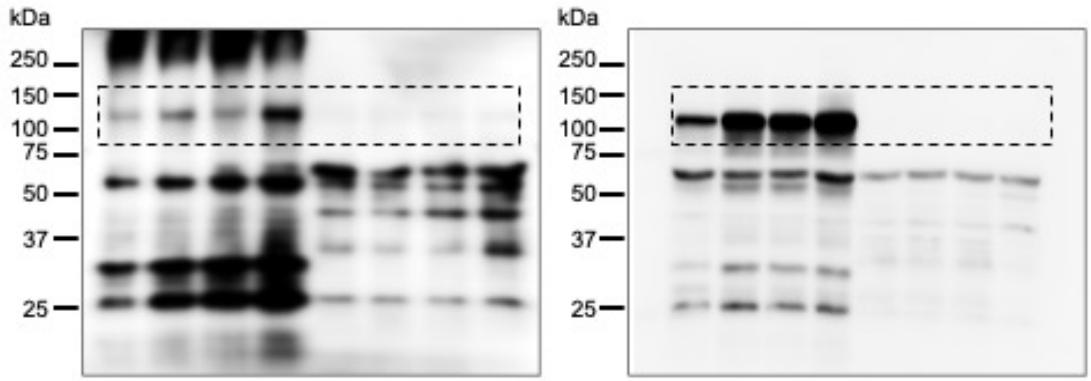

ASC

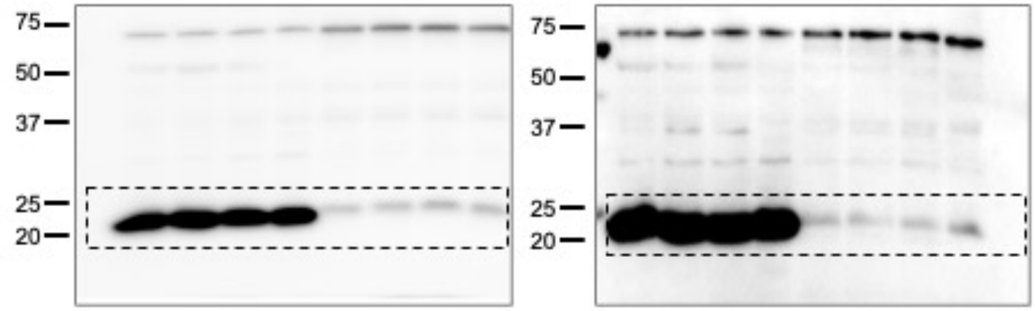

CASP1

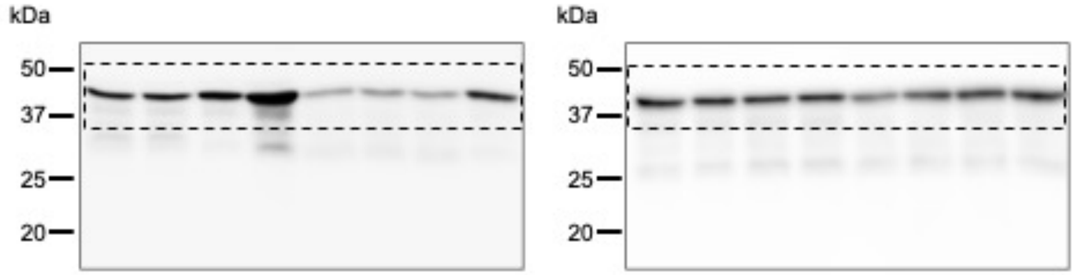

CASP11

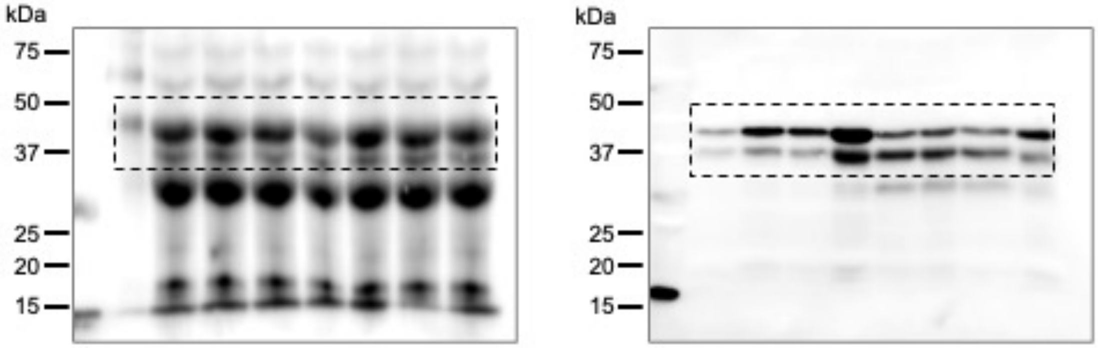

GSDMD

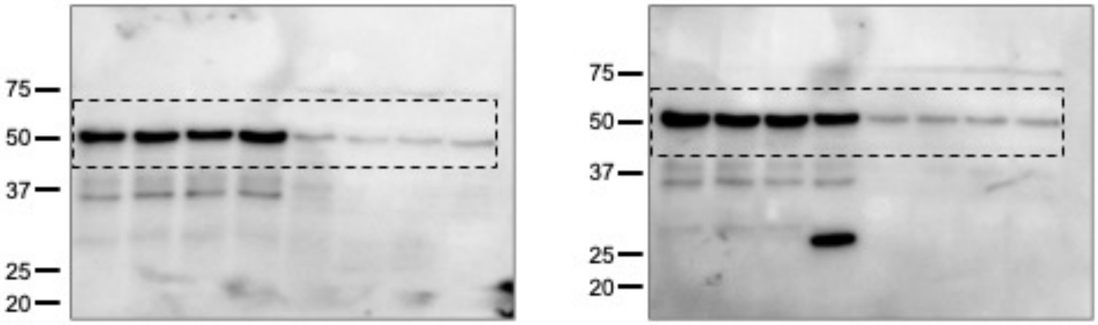

GSDME

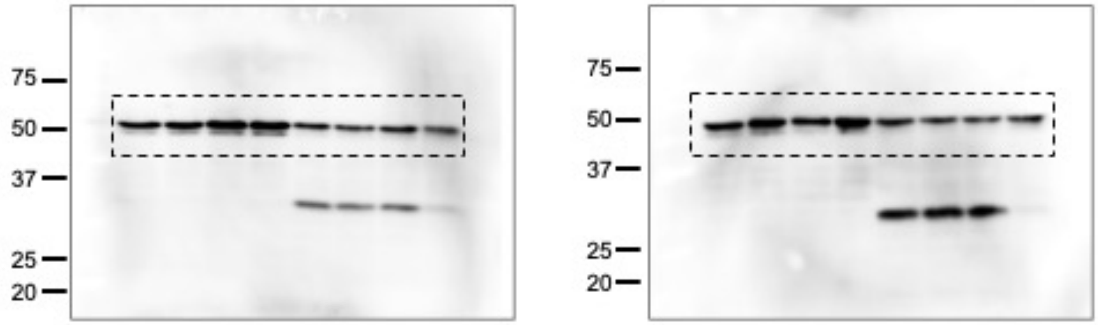

CASP8

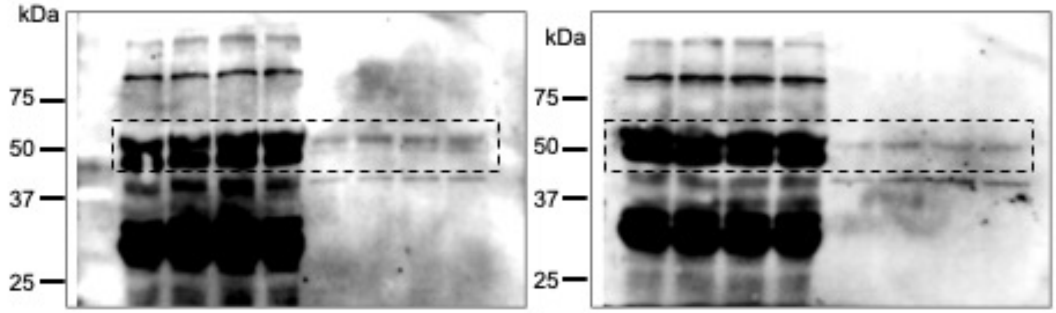

CASP9

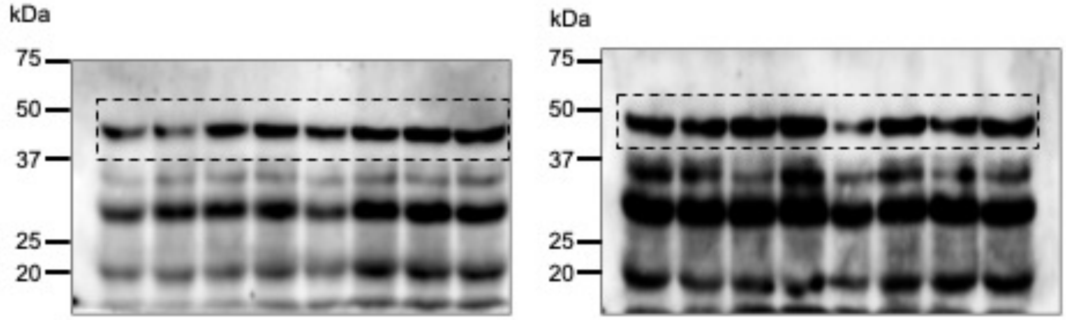

CASP7

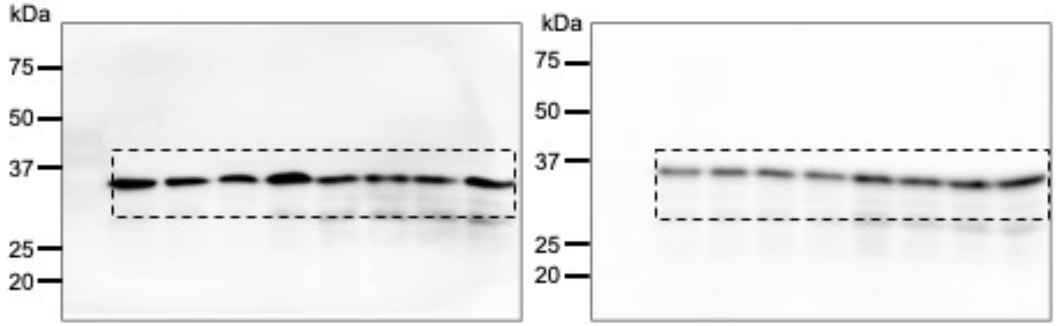

CASP6

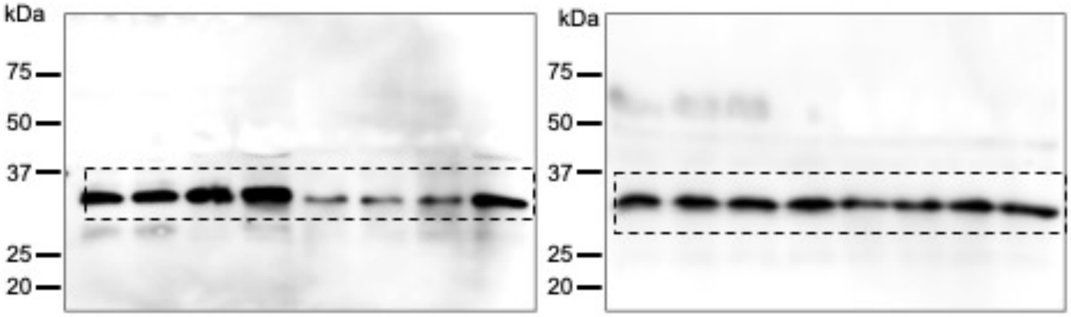

CASP3

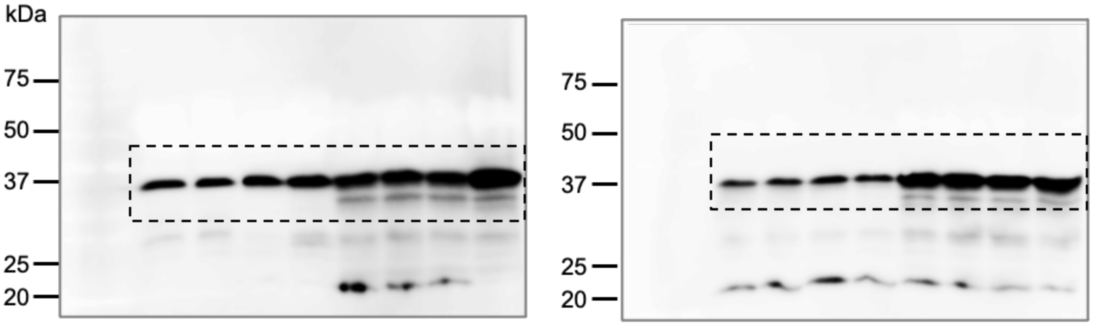

RIPK1

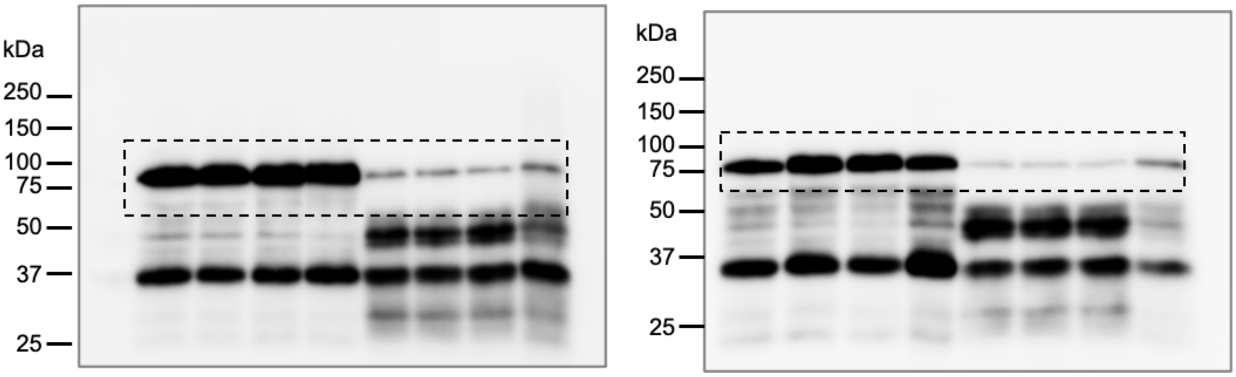

RIPK3

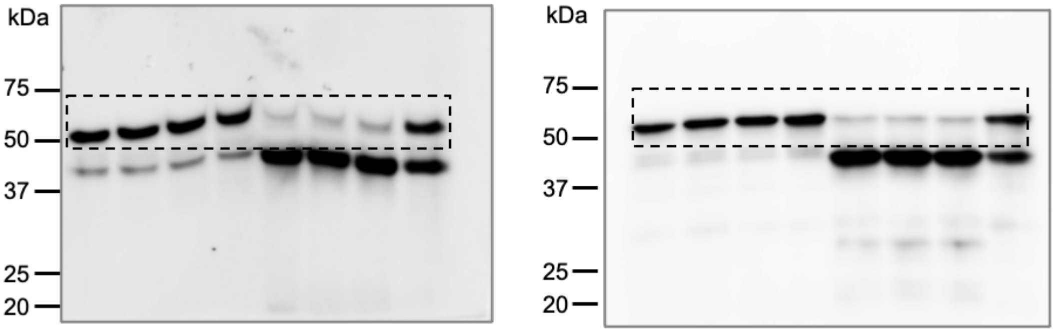

MLKL

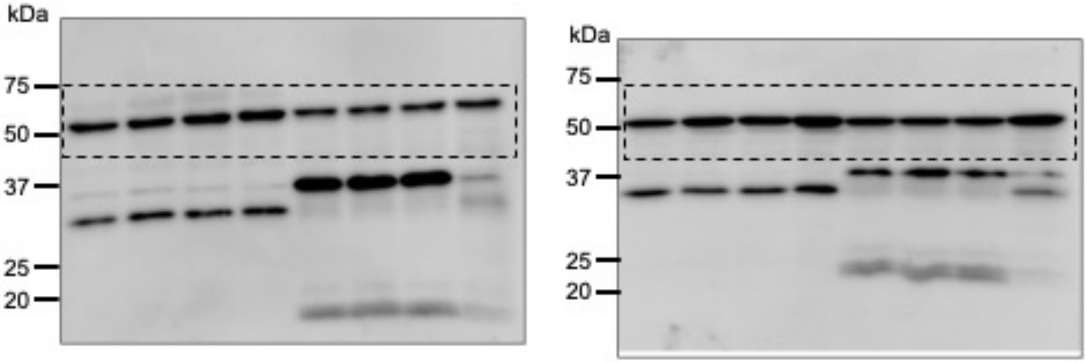

IRF1

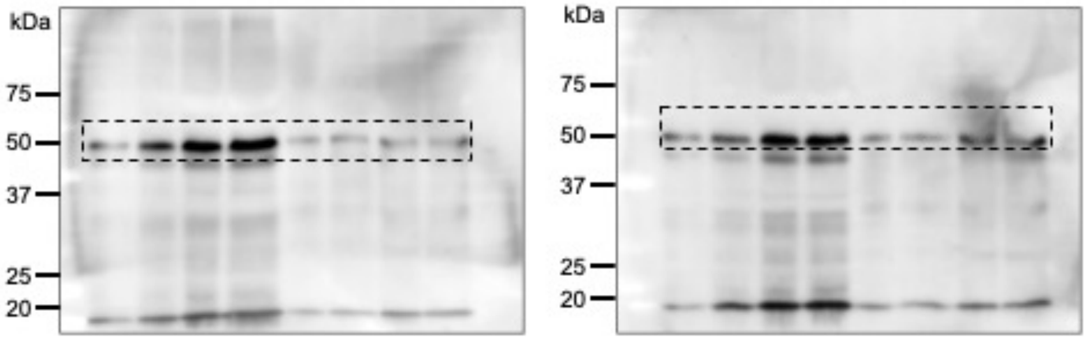

ZBP1

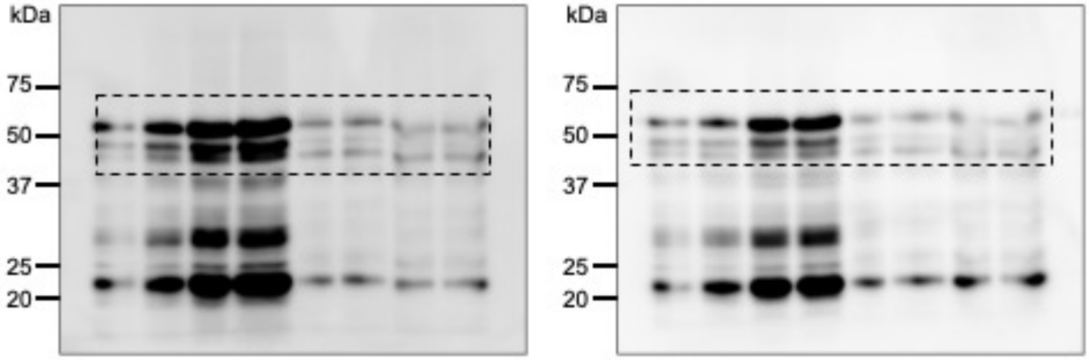

AIM2

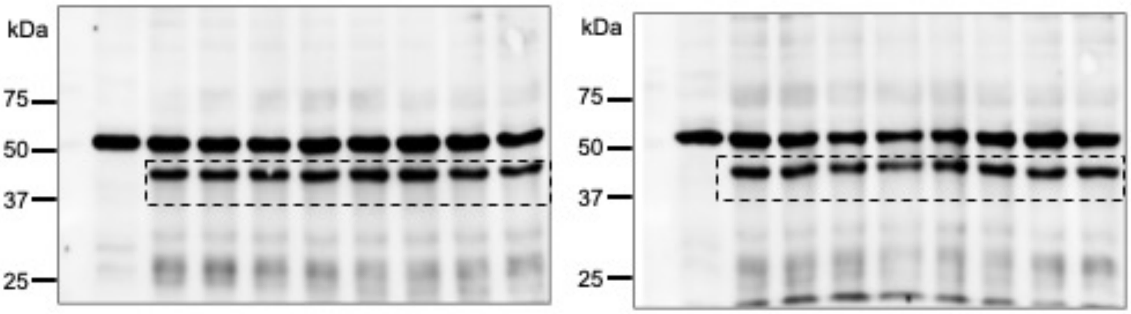

GAPDH

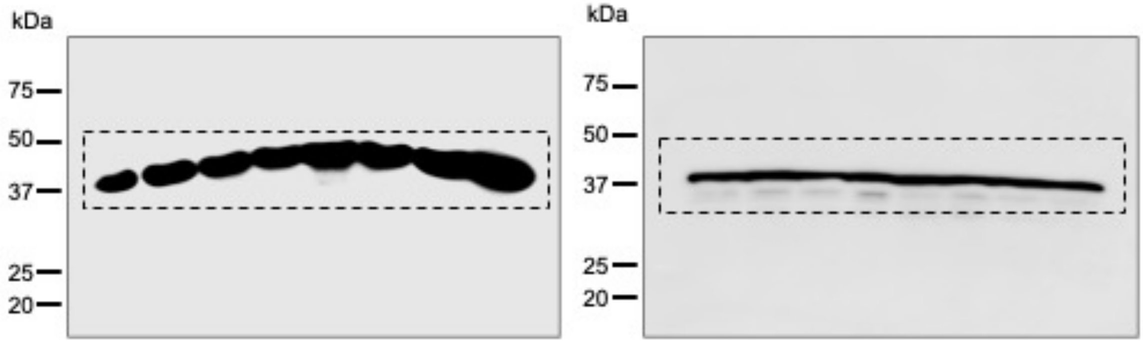

Figure 3 uncropped blots (pages 7-12):

NLRP3

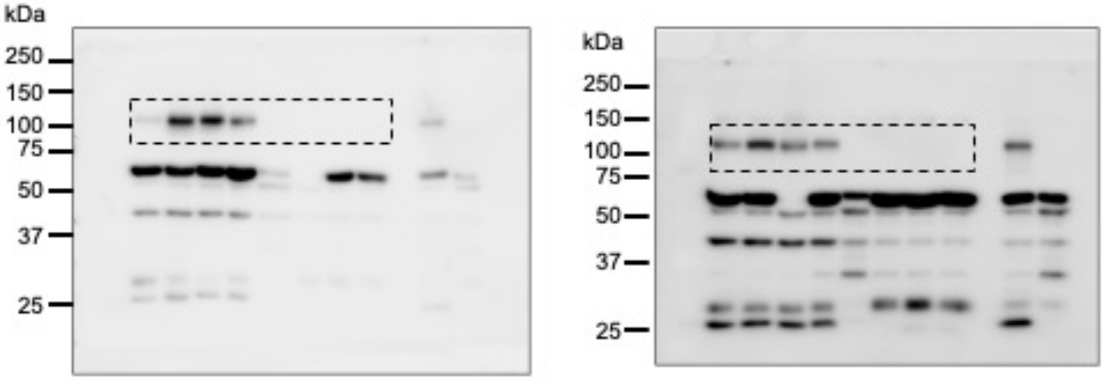

ASC

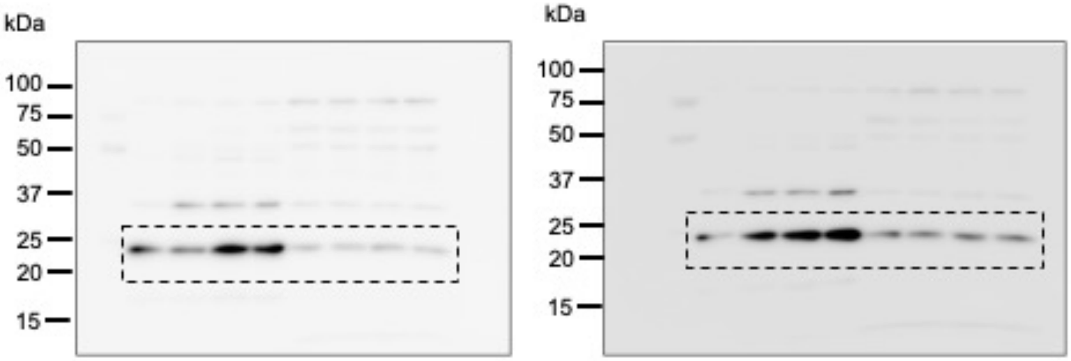

CASP1

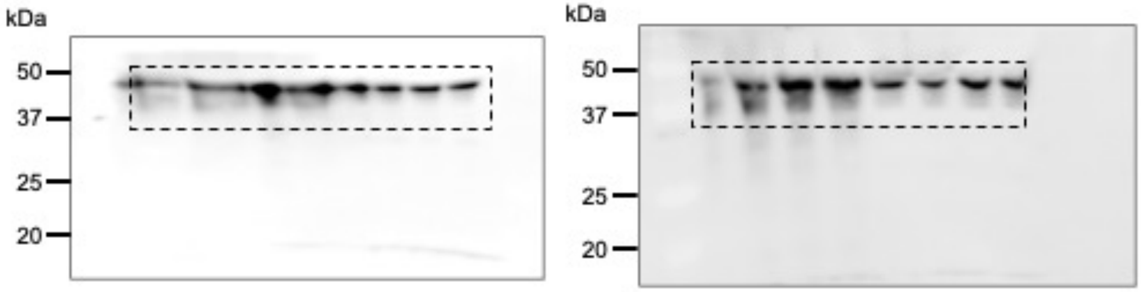

CASP4

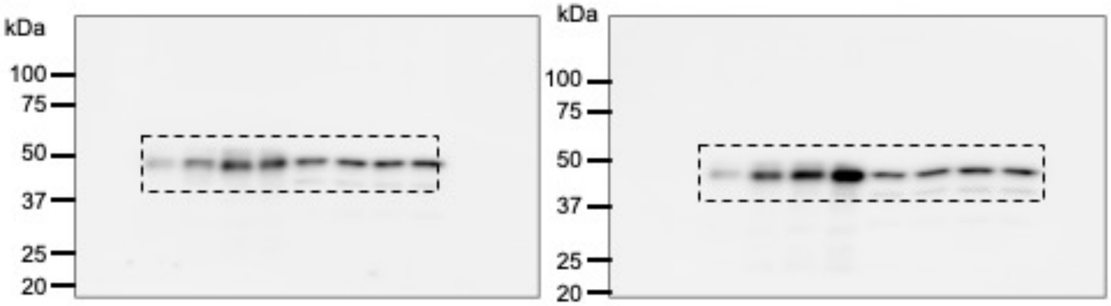

GSDMD

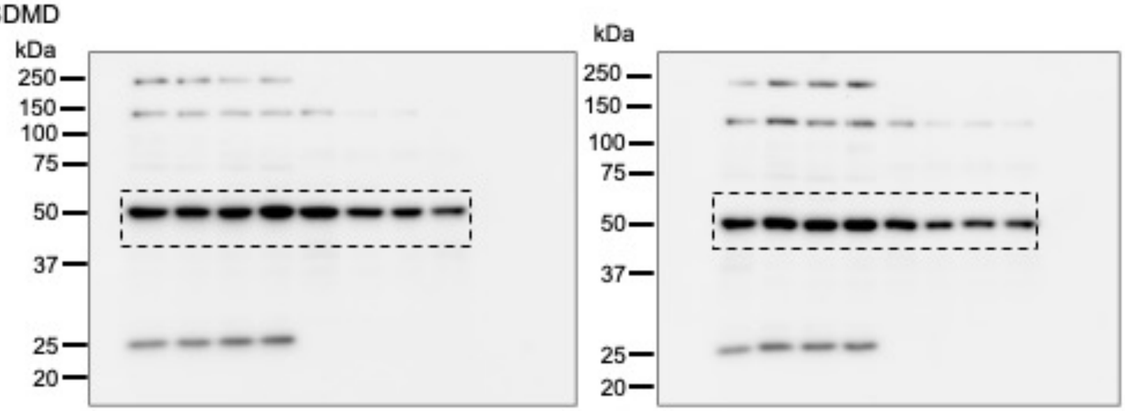

GSDME

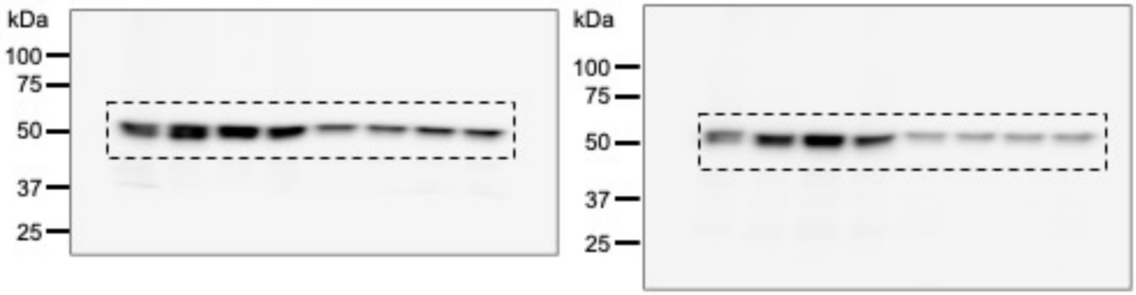

CASP8

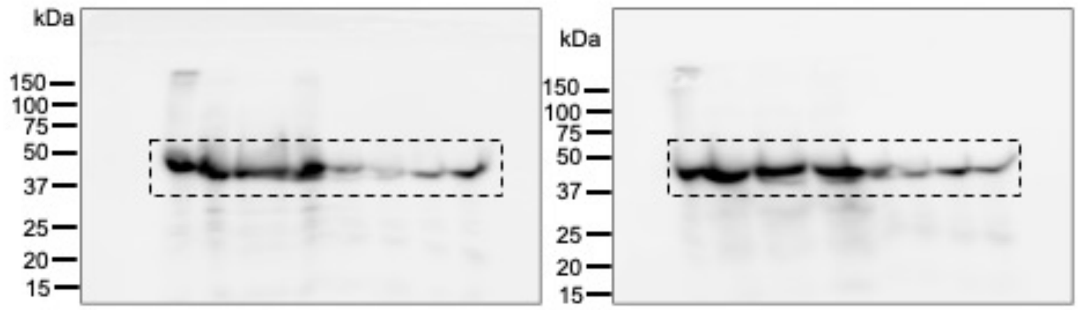

CASP9

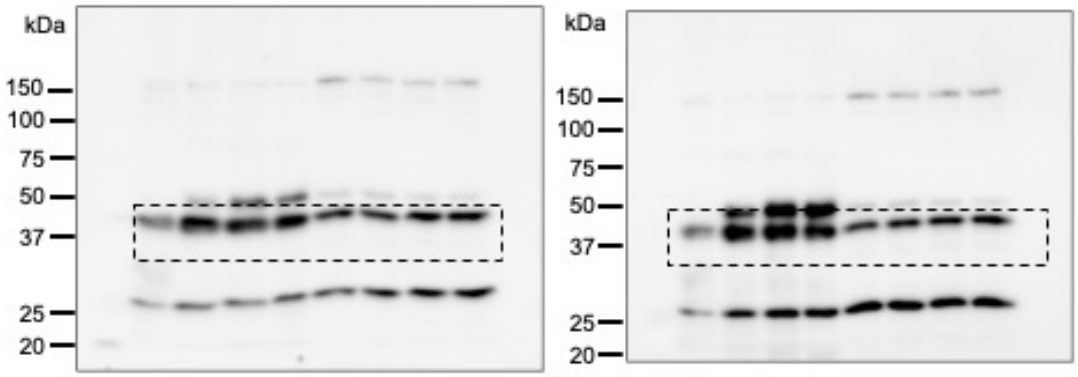

CASP7

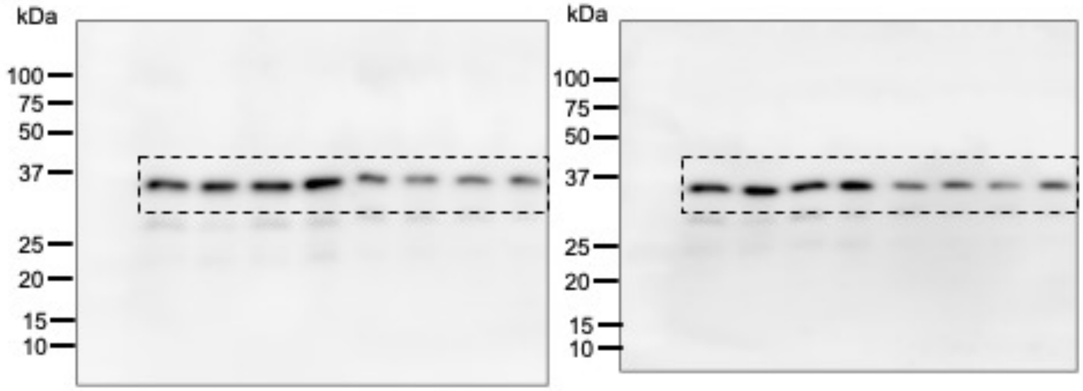

CASP6

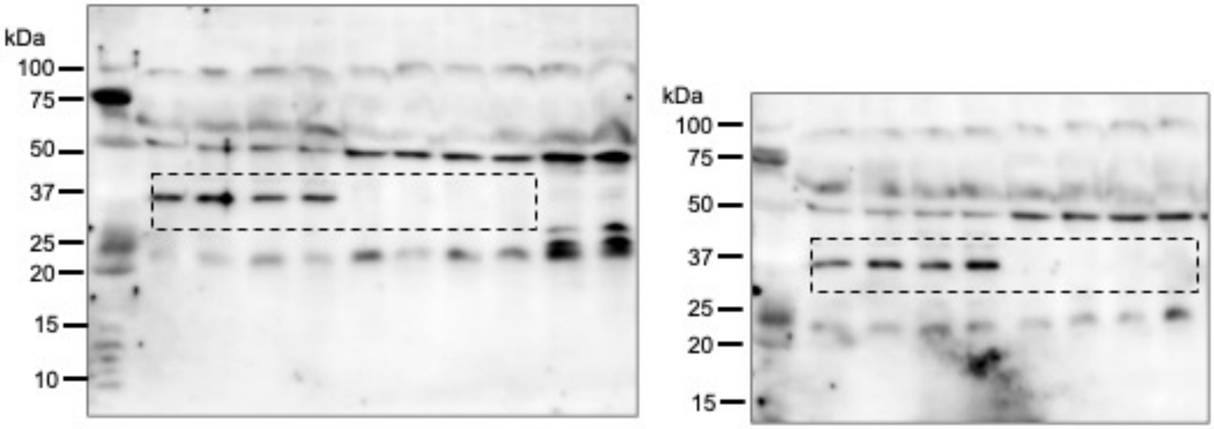

CASP3

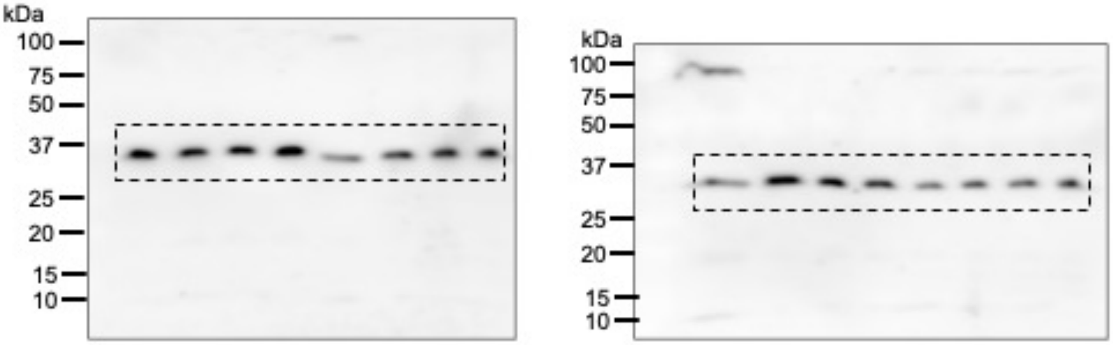

RIPK1

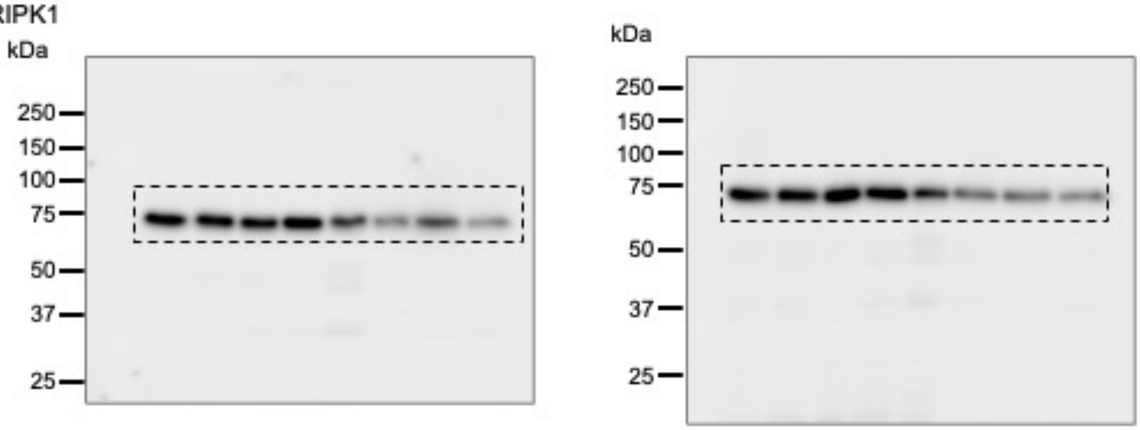

RIPK3

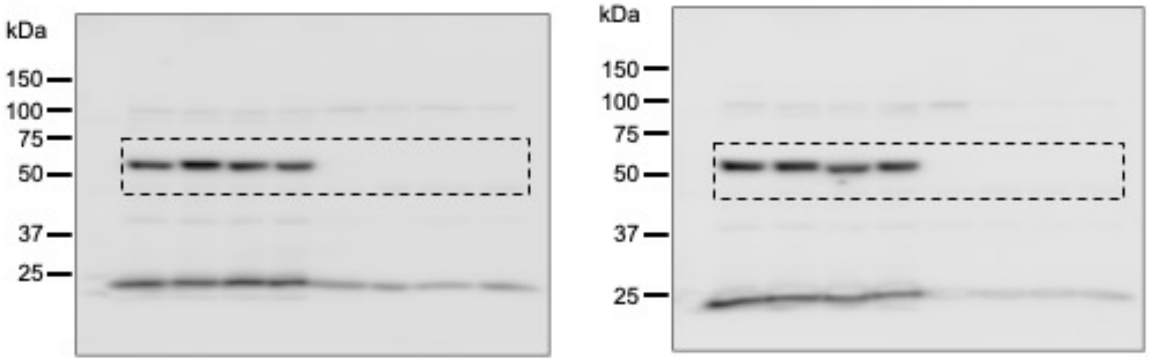

MLKL

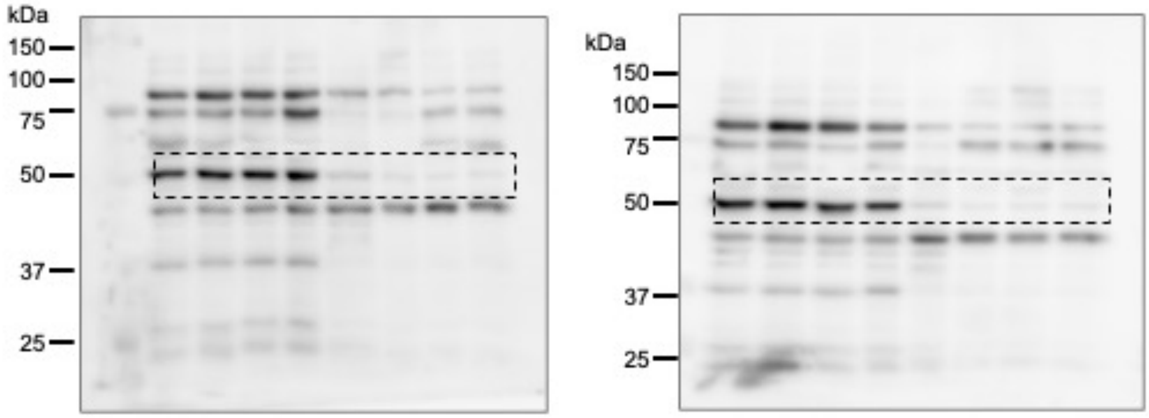

IRF1

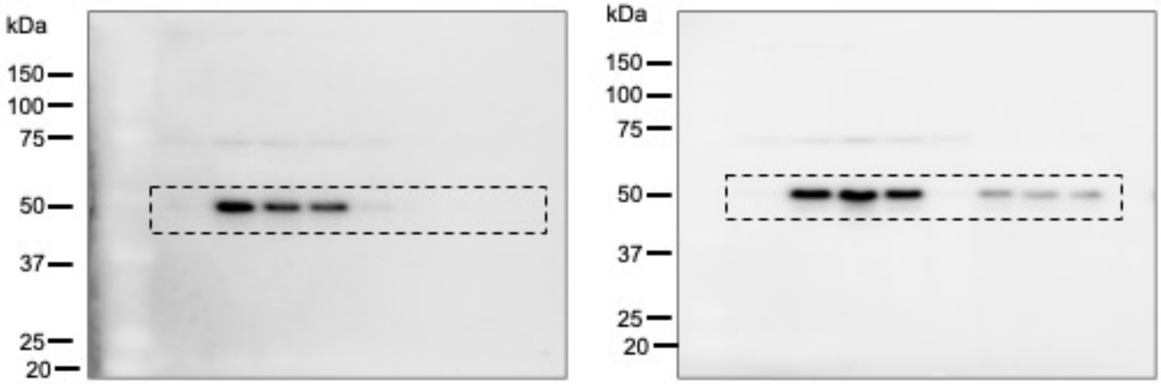

ZBP1

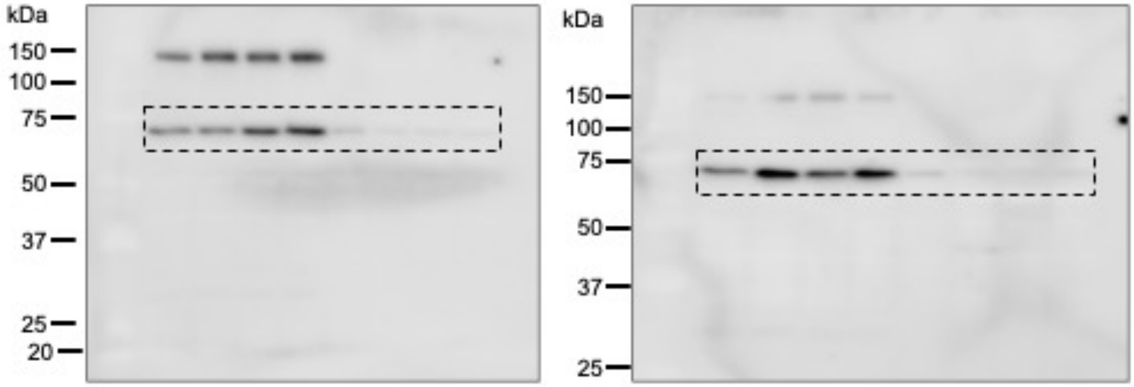

AIM2

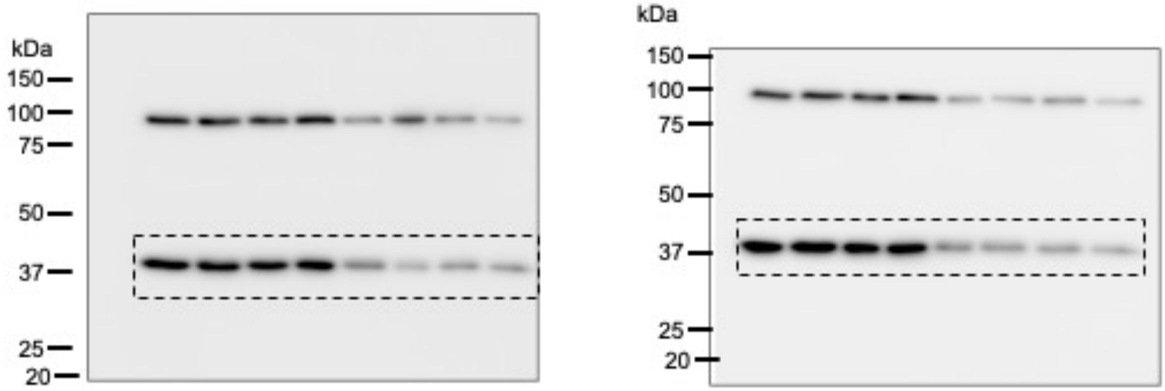

β-actin

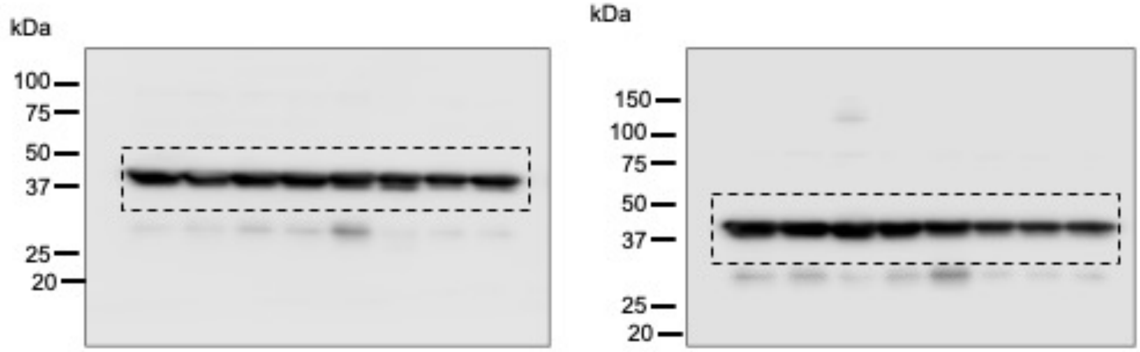

Figure 4 uncropped blots (pages 13-15):

CASP1

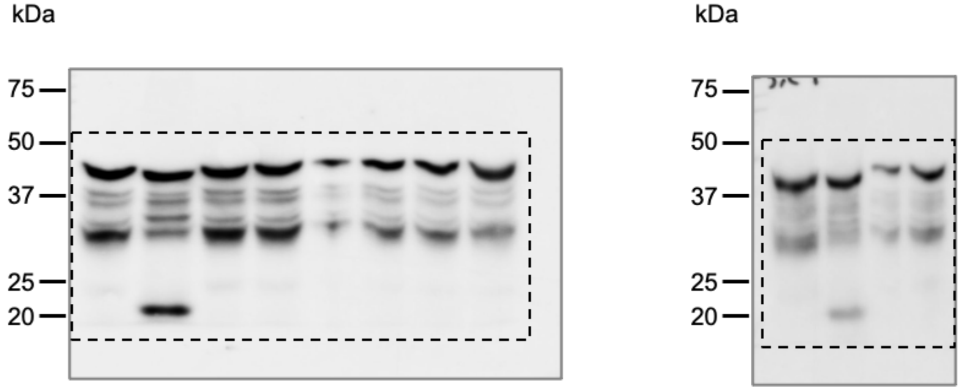

GSDMD

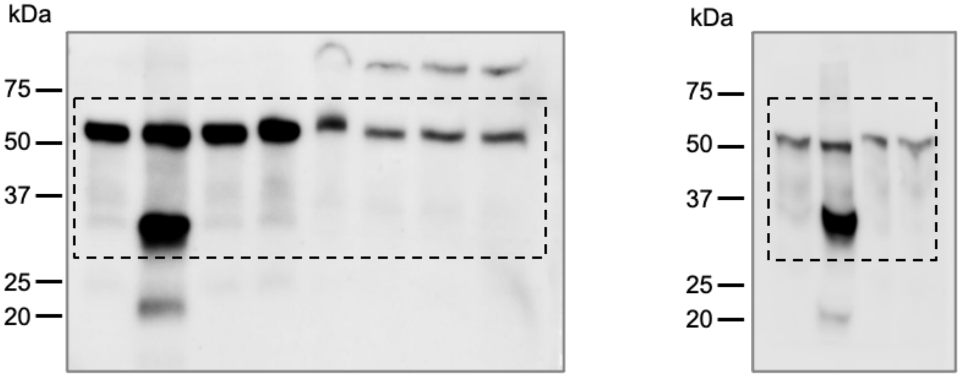

GSDME

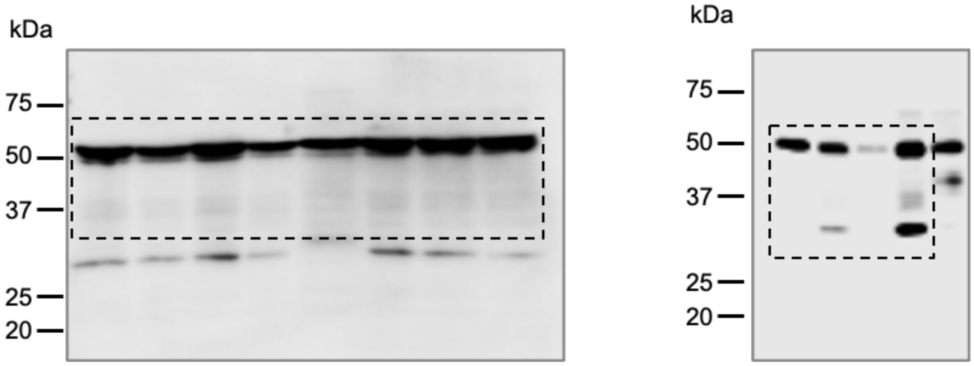

CASP8

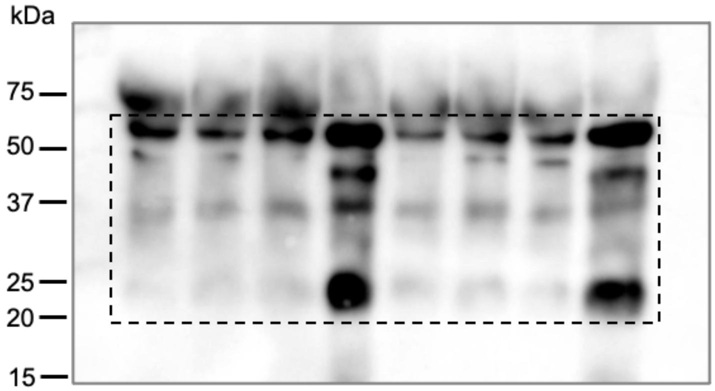

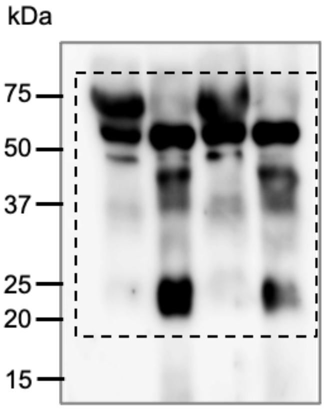

CASP3

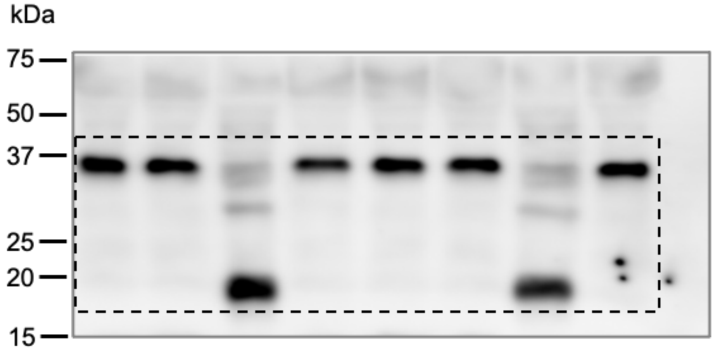

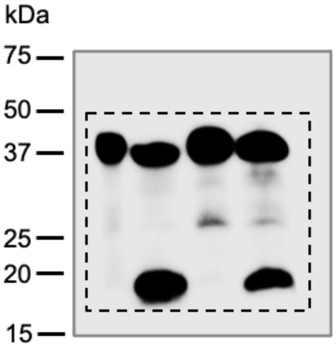

CASP7

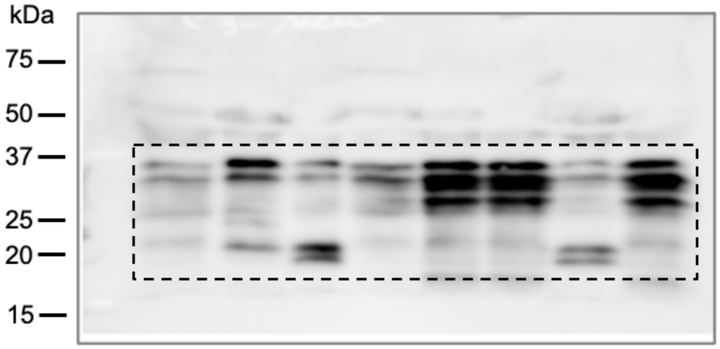

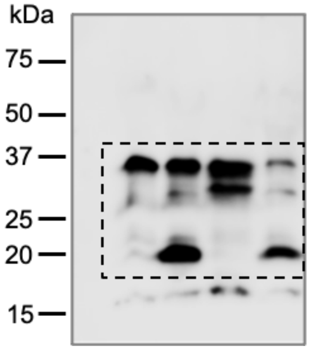

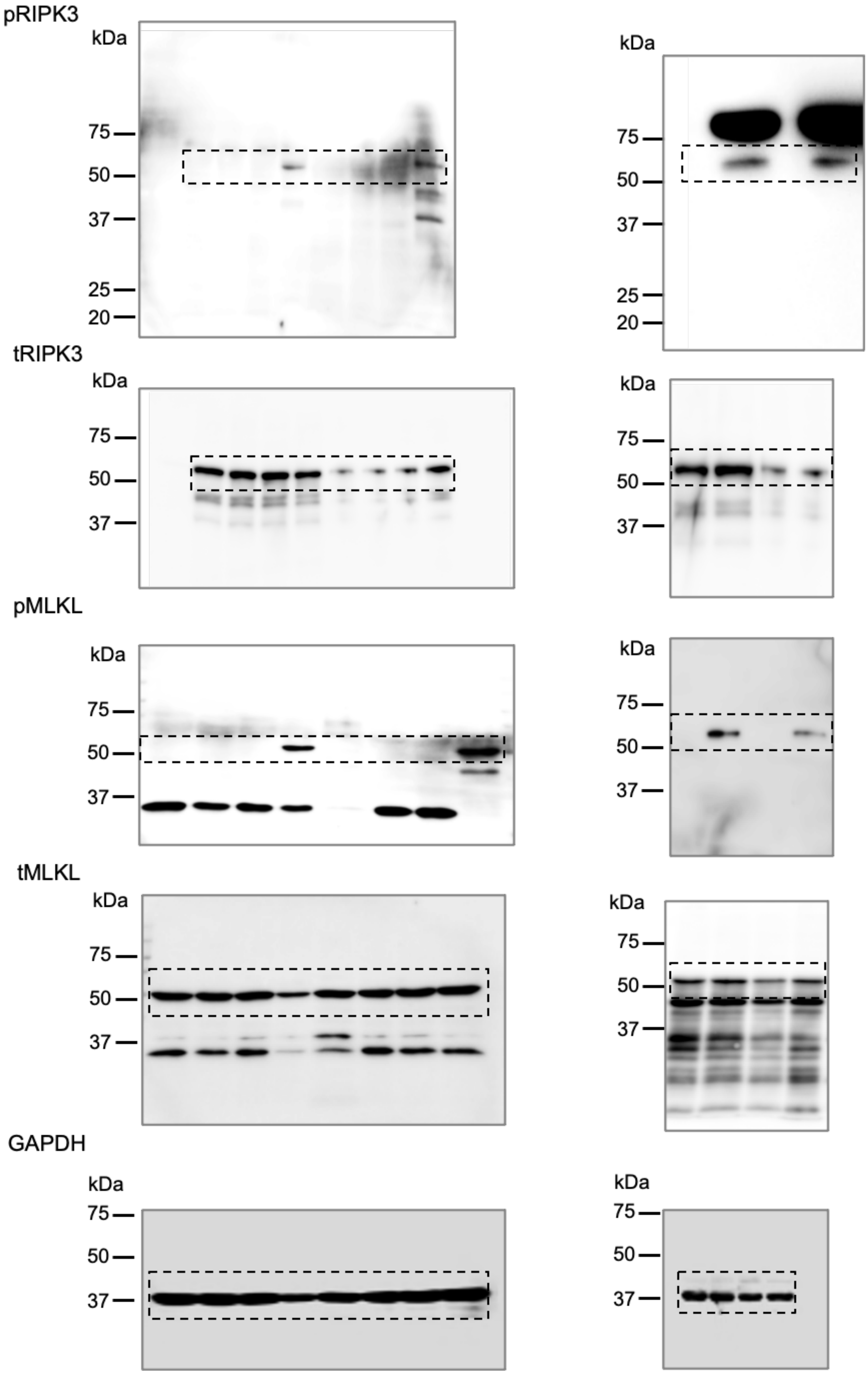
pRIPK3
kDa
75
50
37
25
20
kDa
75
50
37
25
20
tRIPK3
kDa
75
50
37
kDa
75
50
37
pMLKL
kDa
75
50
37
kDa
75
50
37
tMLKL
kDa
75
50
37
kDa
75
50
37
GAPDH
kDa
75
50
37
kDa
75
50
37

Figure 5 uncropped blots (pages 16-18):

CASP1

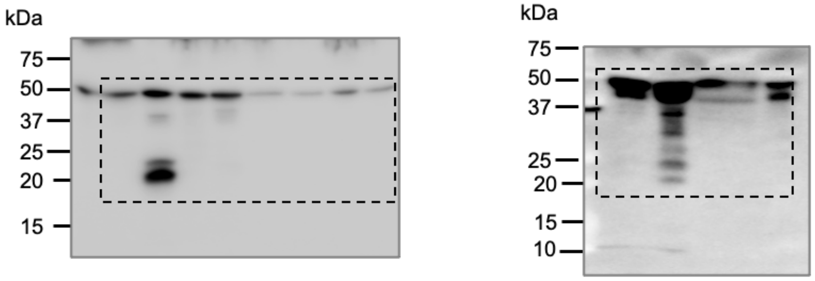

GSDMD

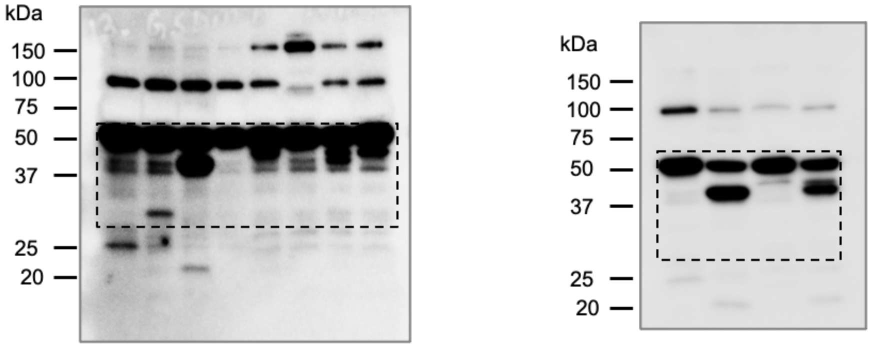

GSDME

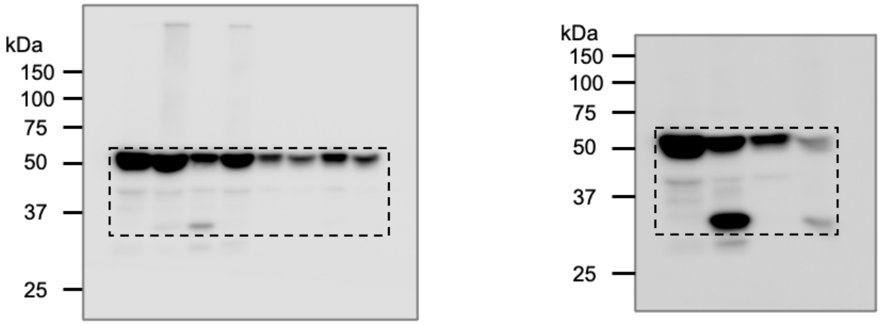

CASP8

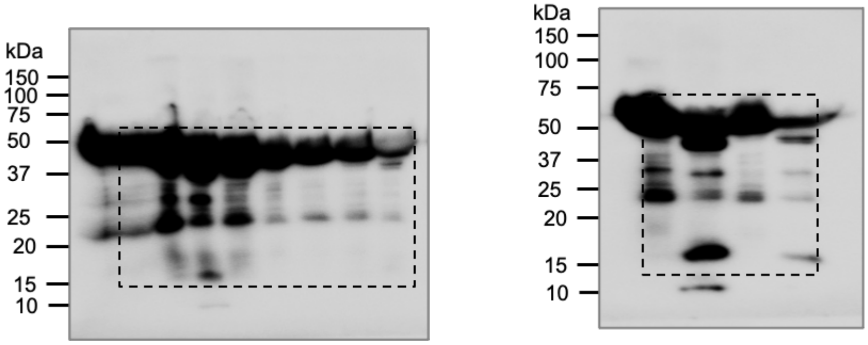

CASP3

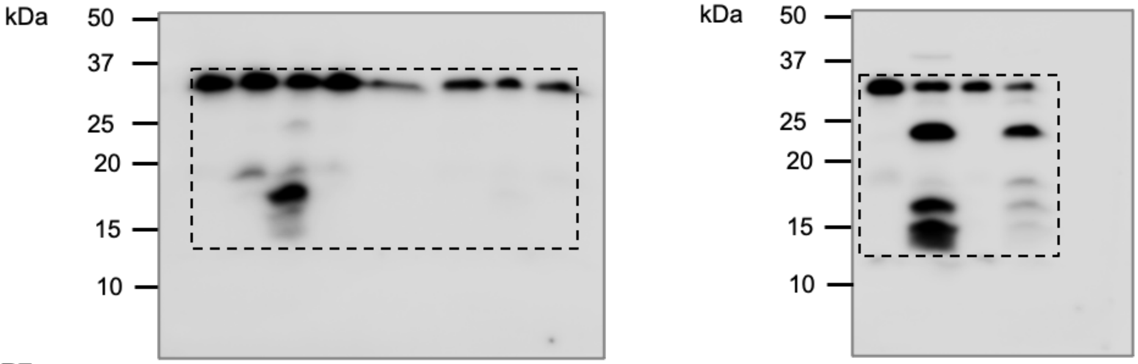

CASP7

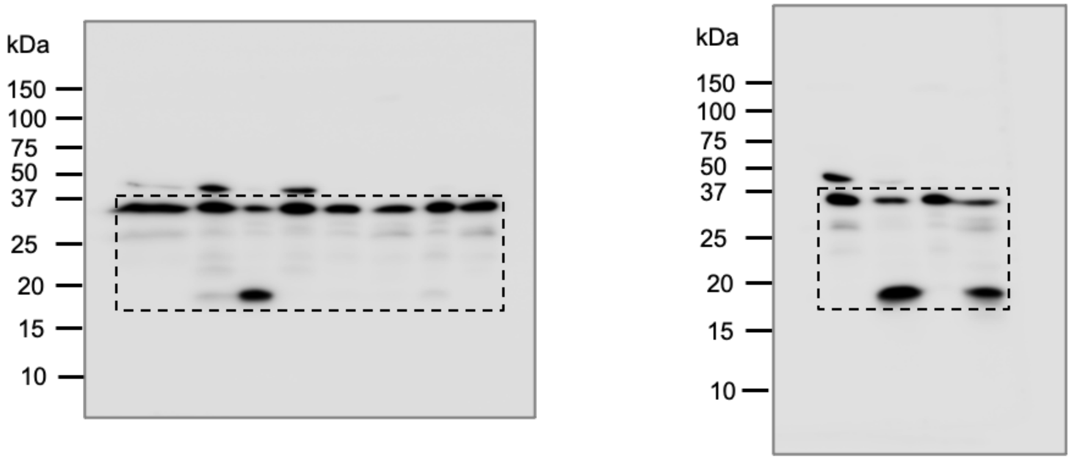

pRIPK3

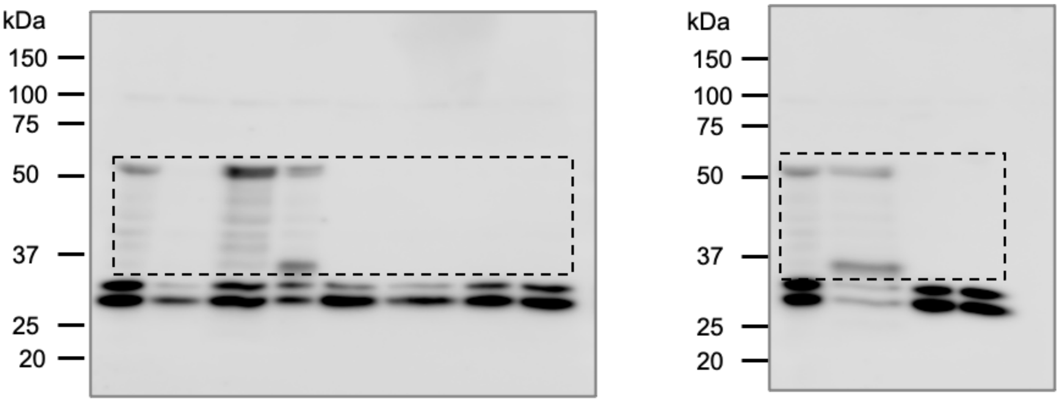

tRIPK3

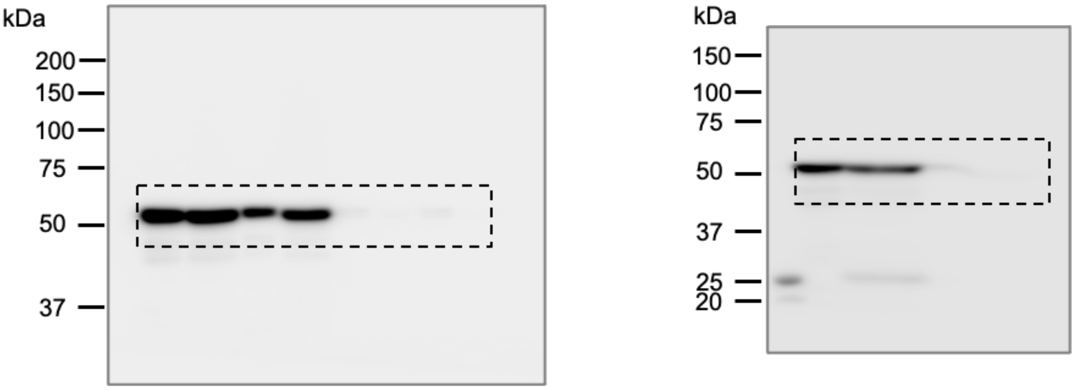

pMLKL

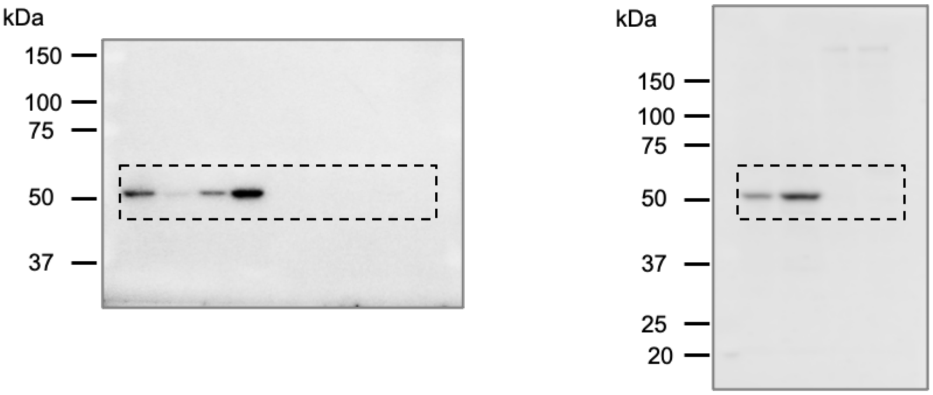

tMLKL

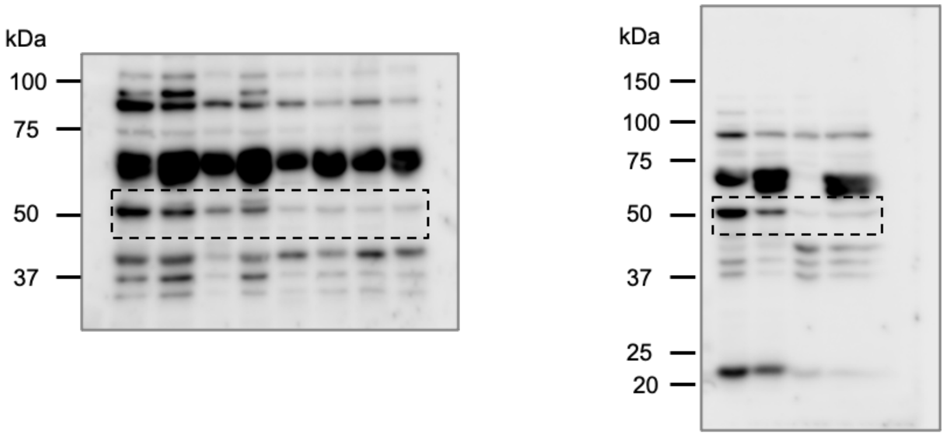

β-actin

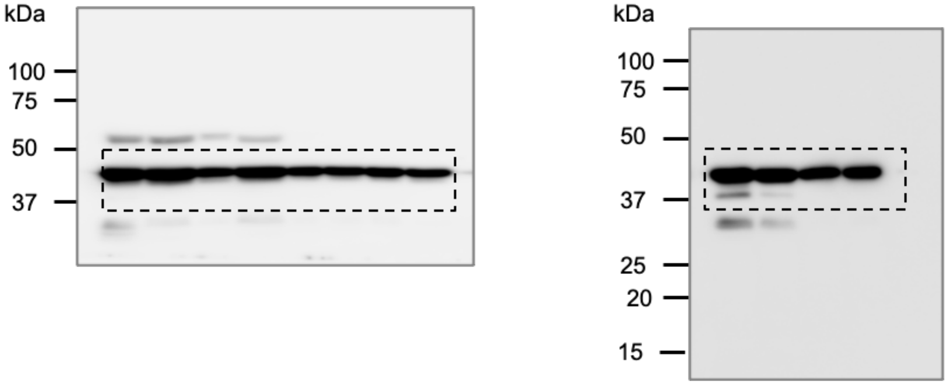

Supplemental Figure uncropped blots (page 19):

Supplemental Figure S3 uncropped blots

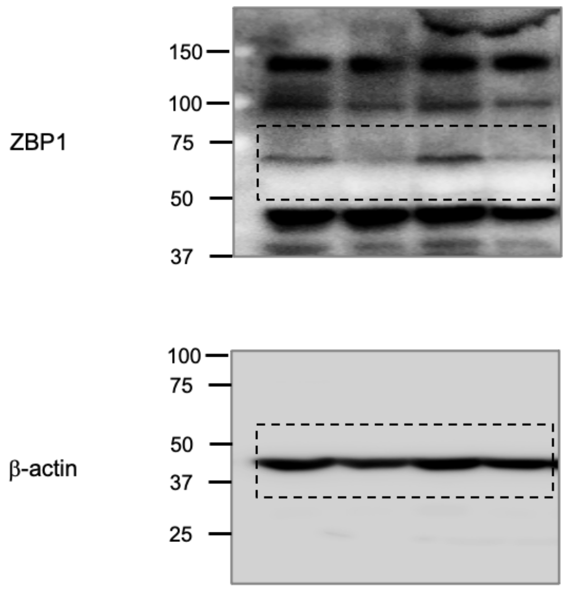

Supplemental Figure S4 uncropped blots

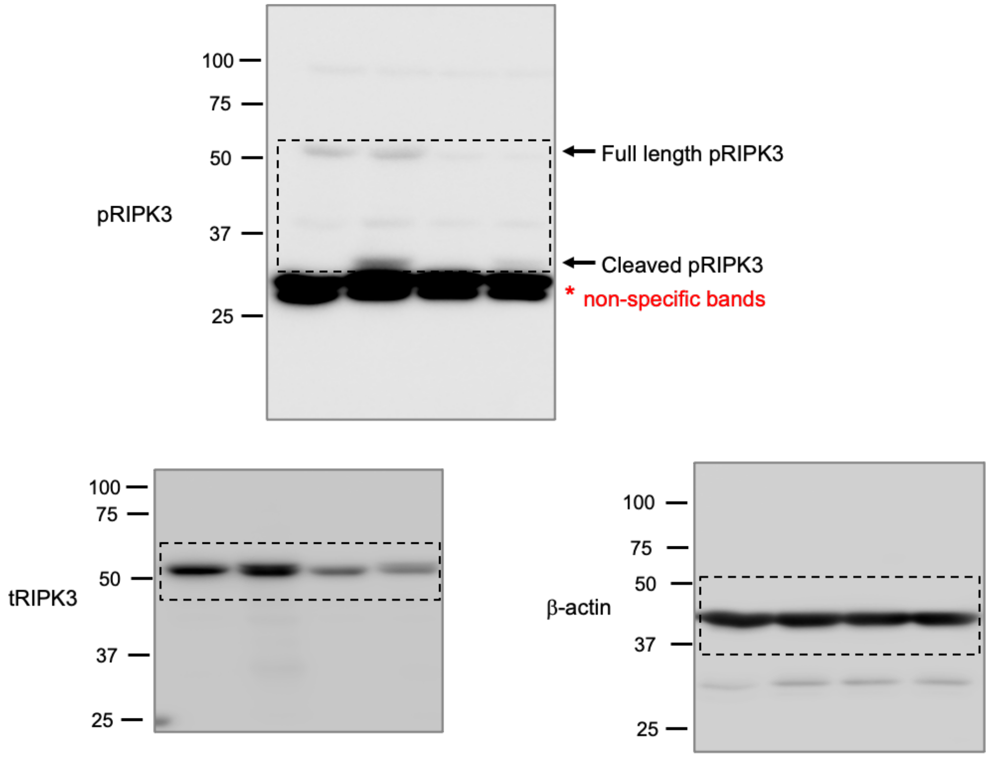

Supplement: S1 Appendix — (PDF) [file pone.0299577.s005.pdf]
